# Genome-wide identification and analysis of *WRKY* gene family in maize provide insights into regulatory network in response to abiotic stresses

**DOI:** 10.1186/s12870-021-03206-z

**Published:** 2021-09-20

**Authors:** Wenjing Hu, Qiaoyu Ren, Yali Chen, Guoliang Xu, Yexiong Qian

**Affiliations:** Anhui Provincial Key Lab. of the Conservation and Exploitation of Biological Resources, Anhui Normal University, Wuhu, 241000 China

**Keywords:** WRKY transcription factor, Expression patterns, Gene duplication, Abiotic stress, Regulatory network analysis, *Zea mays L*

## Abstract

**Background:**

The WRKY transcription factor family plays significant roles in biotic and abiotic stress responses, which has been associated with various biological processes in higher plants. However, very little is known regarding the structure and function of *WRKY* genes in maize.

**Results:**

In this study, a total of 140 ZmWRKY proteins encoded by 125 *ZmWRKY* genes were eventually identified in maize. On the basis of features of molecular structure and a comparison of phylogenetic relationships of WRKY transcription factor families from *Arabidopsis*, rice and maize, all 140 ZmWRKY proteins in maize were divided into three main groups (Groups I, II and III) and the Group II was further classified into five subgroups. The characteristics of exon-intron structure of these putative *ZmWRKY* genes and conserved protein motifs of their encoded ZmWRKY proteins were also presented respectively, which was in accordance with the group classification results. Promoter analysis suggested that *ZmWRKY* genes shared many abiotic stress-related elements and hormone-related elements. Gene duplication analysis revealed that the segmental duplication and purifying selection might play a significant role during the evolution of the *WRKY* gene family in maize. Using RNA-seq data, transcriptome analysis indicated that most of *ZmWRKY* genes displayed differential expression patterns at different developmental stages of maize. Further, by quantitative real-time PCR analysis, twenty-one *ZmWRKY* genes were confirmed to respond to two different abiotic stress treatments, suggesting their potential roles in various abiotic stress responses. In addition, RNA-seq dataset was used to conduct weighted gene co-expression network analysis (WGCNA) in order to recognize gene subsets possessing similar expression patterns and highly correlated with each other within different metabolic networks. Further, subcellular localization prediction, functional annotation and interaction analysis of ZmWRKY proteins were also performed to predict their interactions and associations involved in potential regulatory network.

**Conclusions:**

Taken together, the present study will serve to present an important theoretical basis for further exploring function and regulatory mechanism of *ZmWRKY* genes in the growth, development, and adaptation to abiotic stresses in maize.

**Supplementary Information:**

The online version contains supplementary material available at 10.1186/s12870-021-03206-z.

## Background

Maize is the main source of food security and economic development in sub-Saharan Africa and Latin America, and is among the top three crops in Asia [1]. As a result of the global environmental vagaries, various environmental stresses including biotic and abiotic stresses have brought huge threat to the global maize production [2, 3]. Currently, abiotic stresses, such as extreme temperature, high salinity and drought and so on, have been confirmed to be one of the main factors for the losses of corn yield worldwide. For example, it has been estimated that a one-degree temperature rise reduces maize yields around the world by 7.4% [4]. Therefore, it is emergent to clarify the molecular mechanism of maize in response to abiotic stresses.

The WRKY TF family is one of the largest transcription factor (TF) families in higher plants [5]. A number of studies have demonstrated that the WRKY transcription factors play critical roles in response to biotic and abiotic stress [6]. The WRKY proteins can induce or repress the expression of their downstream genes by specifically binding to W-box [TGACC (A/T)] at their promoter sites and eventually activate their stress responses [7]. One of the distinguishing features of the WRKY TFs is the presence of highly conserved WRKY domain. The conserved WRKY domain is composed of approximately 60 amino acid residues with a highly conserved heptapeptides, WRKYGQK, at the N-terminus, and a novel zinc finger motif C_2_H_2_ (C–X_4–5_–C–X_22–23_–H–X–H) or C_2_HC (C–X_7_–C–X_23_–H–X– C) at their C-terminus [8–10]. On the basis of the characteristics of WRKY domain and zinc-finger-like motif, the WRKY TFs can be grouped into three major Groups (I, II, and III). The Group II WRKY members are further divided into five subgroups (IIa-IIe) according to their evolutionary divergence [8, 11]. The Group I members contain two WRKY domains with C_2_H_2_ zinc-finger-like motifs. The Group II members contain a single WRKY domain including a C_2_H_2_ zinc-finger-like motif. The WRKY TFs containing a single WRKY domain with a C_2_HC zinc-finger-like motif belong to the Group III [8].

It has been reported in a great deal of studies that the *WRKY* genes respond to specific abiotic stresses, such as drought, waterlogging, wounding, and salt stress [12–14]. For example, *AtWRKY30* can be induced under various abiotic stresses, meanwhile, the overexpression of *AtWRKY30* greatly enhance the resistance of *Arabidopsis* in response to salt stress [15]. Overexpression of *OsWRKY47* can raise the rice production, and the tolerance of rice to drought also can be improved compared with normal rice plants [16]. Additionally, a great amount (54/103) of *OsWRKY* genes showed remarkable changes in expression levels after salt, drought and cold stresses treatments [17]. Moreover, in wheat, the majority (8 out of 15) of *TaWRKY* genes were transcribed in response to cold, heat, salt and PEG treatments [18].

As far as we know, only few *WRKY* genes have been reported in maize. *ZmWRKY17* could control the transcription of some stress- and ABA-related genes, and eventually improved the salt stress resistance and reduced ABA sensitivity [19]. *ZmWRKY33* could be activated by some abiotic stress such as high-salt, dehydration, cold, and ABA treatments, and it increased the salt stress resistance of transgenic *Arabidopsis* [20]. In addition, *ZmWRKY58* enhanced both salt and drought stress tolerance of transgenic rice [21]. These studies demonstrated that the role of *ZmWRKY*s in terms of enhancing tolerance to abiotic stresses. Although over 100 members of the maize *WRKY* gene family have been proposed [22], the expression patterns of *ZmWRKY*s in different tissues of maize under abiotic stresses have not been investigated at genome-wide level. Many details about maize *WRKY* gene family remain to be further elucidated.

In this study, 140 WRKY TF proteins were identified from the latest B73 maize genome database and orderly named. Moreover, a comprehensive analysis of *ZmWRKY* genes was accomplished including their phylogenetic relationship, chromosome location, gene duplication, exon-intron structure, cis-acting factors, conserved domains and expression patterns in various tissues and under salt and drought stress treatments. Furthermore, weighted gene co-expression network analysis was used to identify modules of co-expression network and explore key genes involved in plant development. Further, subcellular localization and Gene ontology (GO) annotation were performed using the online WOLF PSORT software and Blast2GO software to analyze functional classification of ZmWRKY proteins in maize respectively and PPI (protein-protein interaction) network was constructed using the Search Tool for the Retrieval of Interacting Gene (STRING) database to further understand the biological and molecular functions of ZmWRKY proteins. Taken together, these results may provide an in-depth understanding of the evolution of *WRKY* gene family in maize and their critical roles played in abiotic stress responses.

## Results

### Identification and analysis of WRKY genes in maize

In this study, a set of 140 WRKY TF proteins were identified through the maize genomic database (Table S1). A total of 125 *WRKY* genes were consistently named as *ZmWRKY1*- *ZmWRKY125* based on their chromosome location, while the variant proteins generated from the same locus were granted by the same name followed by 1, 2 or 3. The characteristics were analyzed including the genome location of these identified genes, length of the open reading frame (ORF), basic information of their encoded proteins including length, molecular weight (MW) and isoelectric point (pI). As shown in the Table S1, the length of ZmWRKY proteins varied from 99 (ZmWRKY7) to 729 (ZmWRKY59) amino acids and the average protein sequence length is 349 residues. The pI ranged from 4.5514 (ZmWRKY98) to 10.7787 (ZmWRKY125), and the MW (molecular mass) ranged from 11,218.7 Da (ZmWRKY7) to 78,734.7 Da (ZmWRKY59).

### Classification of maize WRKY proteins

**Fig. 1:**
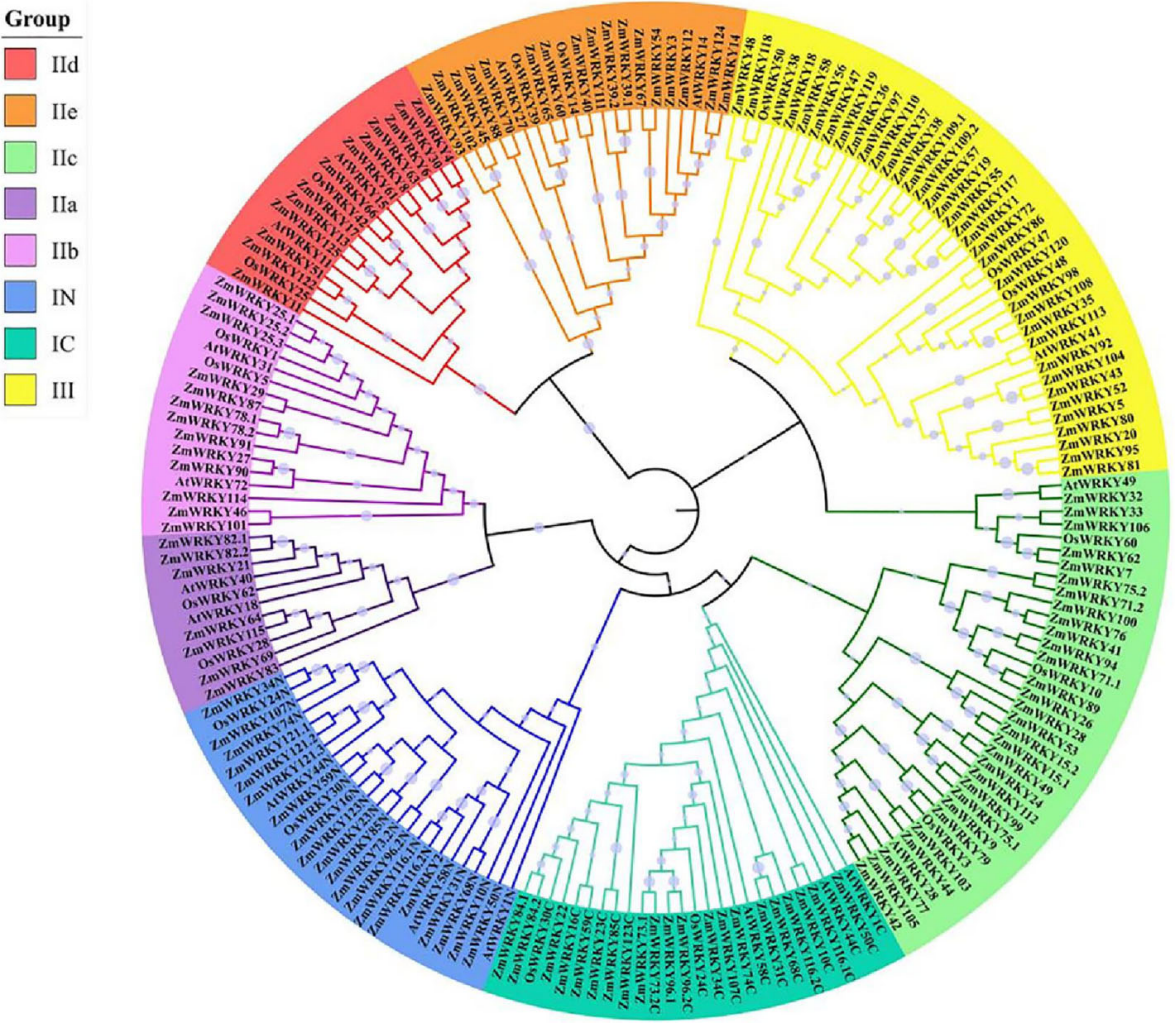
Phylogenetic analysis among WRKY domains of WRKY proteins of maize, rice and *Arabidopsis*. The Unrooted neighbor-joining (NJ) phylogenetic tree was constructed with WRKY domains of WRKY proteins from maize, rice and *Arabidopsis* using MEGA7.0 with a bootstrap of 1000. Gray circles of different sizes represent the level of support. Eight main clades are marked: IN, IC, IIa, IIb, IIc, IId, IIe, and III with different colored ranges. Protein sequences were downloaded from National Center for Biotechnology Information (NCBI) and Maize genome database. The respective WRKY domain sequences of candidate WRKY proteins were downloaded from the Smart database

To analyze the phylogenetic relatedness among ZmWRKY proteins in maize, a total of 156 conserved WRKY domains including two WRKY domains of the Group I members, were extracted to construct the evolutionary tree using the neighboring method. To obtain a more precise result, 16 OsWRKY proteins from rice (*O. sativa japonica*) and 15 AtWRKY proteins from *Arabidopsis* were analyzed together with all ZmWRKY proteins identified in this study. The WRKY domain sequences of candidate rice and *Arabidopsis* WRKY proteins were downloaded from the Smart database (http://smart.emblheidelberg.de/). As depicted in the constructed evolutionary tree (Fig. 1), the 140 ZmWRKY proteins were classified into three main Groups (I, II and III), and the Group II proteins were further categorized into 5 subgroups (IIa, IIb, IIc, IId and IIe). To reveal how conservative the heptapeptide WRKYGQK and zinc-finger-like domains were in each group, the sequence logos were produced by the WebLogo online program to exhibit the conservation at each residue position, and the sequence alignment were further performed by DNAMAN 7.0 (Fig. S1). The WRKY TFs have two standard motifs, and the first one WRKYGQK sequence can combine with the W box cis-element to induce their downstream gene expression. Besides the WRKYGQK sequence, three variants, WKKYGQK (ZmWRKY7 and − 62) and WRKYGKK (ZmWRKY26, − 41, − 71.1, − 71.2, − 76, − 89, − 94 and − 100) in the Group IIc and WRKYGEK (ZmWRKY1, − 19, − 55, − 57, − 97 and − 117) in the Group III, were also revealed in the *ZmWRKY* gene family. The other one was a zinc-finger-like domain with two types, namely C_2_H_2_ and C_2_HC.

As shown in Fig. S1, it is worth noting that the Group I was clustered by 25 WRKY proteins, among which there were sixteen members containing two WRKY domains. Fifteen of these proteins contained two intact WRKY domains, however ZmWRKY116.1 only contained a single intact WRKY domain in its N-terminal, while its C-terminal domain lacked a zinc-finger domain. In addition, the other nine members had only a single WRKY domain at either the C-terminal (ZmWRKY22, − 73.1, − 84.1, − 84.2 and − 96.1) or the N-terminal (ZmWRKY2, − 121.1, − 121.2, − 121.3) of these proteins*,* suggesting that they probably had gone through domain loss or acquisition events during their evolutionary process [23]. Moreover, the zinc-finger motifs of the ZmWRKYs in the Group I belonged to the C_2_H_2_ type with a C-X_4_-C-X_22–23_-H-X_1_-H motif (Fig. S1). There were 80 members assigned to the Group II, while 75 of them contained the motif of C-X_4–5_-C-X_23–24_-H-X_1_-H, and three members, ZmWRKY33, − 87 and − 114, lacked a typical zinc-finger-like motif. All the Group II 80 members were further divided into five subgroups according to their phylogenetic relationship. The subgroups were distributed as the follows: the Group IIa (7 proteins), the Group IIb (13 proteins), the Group IIc (30 proteins), the Group IId (13 proteins), and the Group IIe (17 proteins). The zinc-finger motifs of the Group III members (35) belonged to the C_2_HC type, with the C-X_5–7_-C-X_23–38_-H-X_1_-C motif (Fig. S1), except ZmWRKY48 and − 117 only containing fragmentary zinc-finger structure.

### Analyses of chromosomal location, gene duplication and genome synteny

**Fig. 2:**
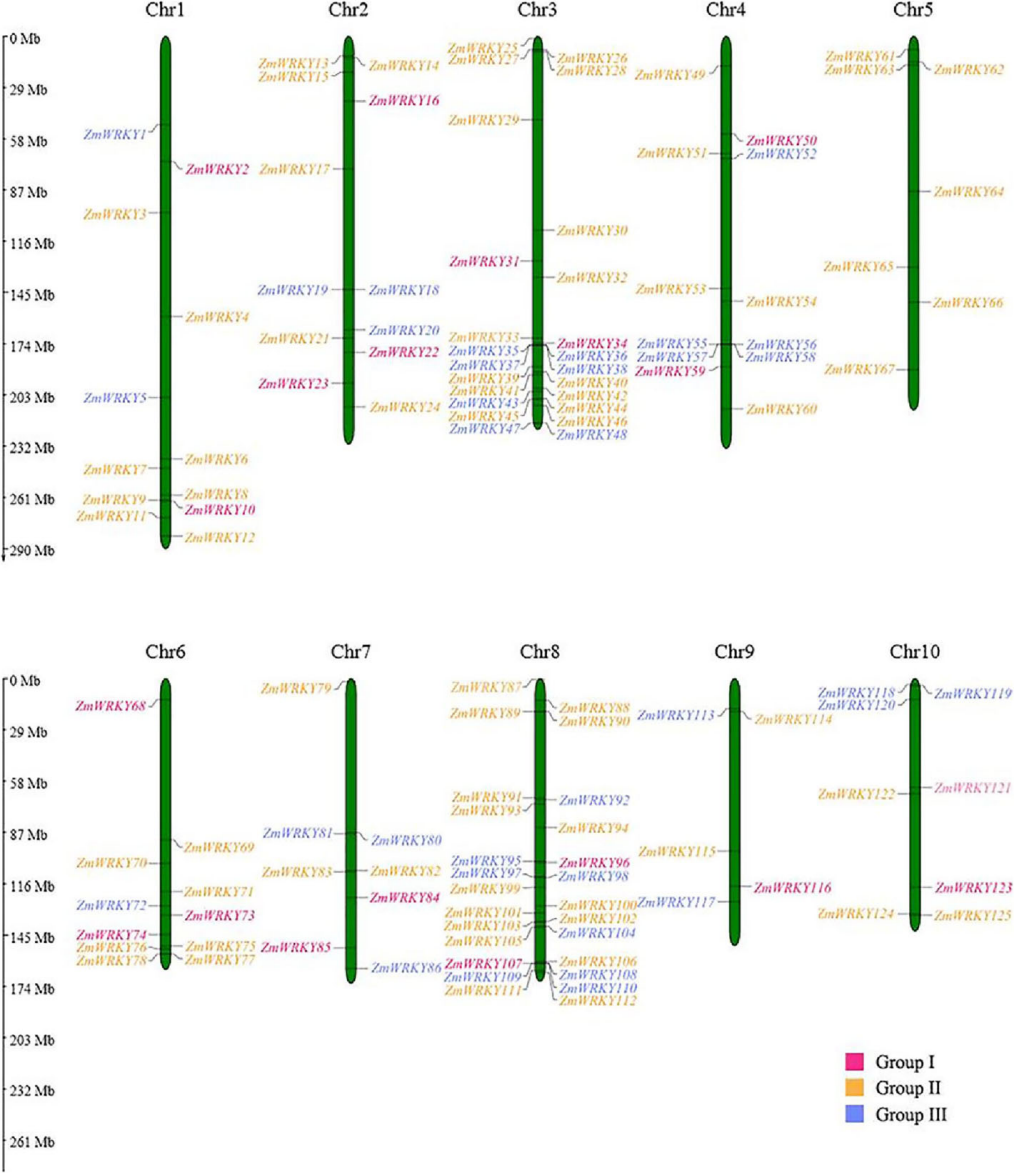
Chromosomal location of *WRKY* family genes in maize. Distribution of *WRKY* genes in maize chromosomes. A total of 125 *ZmWRKY* genes were mapped on the ten maize chromosomes with an uneven distribution. The chromosome numbers are indicated at the top of each vertical gray bar. The gene names on the both side of each chromosome correspond to the approximate locations of each *ZmWRKY* genes. The scale on the left is in megabases

The genomic distribution of Zm*WRKY* genes in maize was carried out by MapInspect software. A total of 125 candidate *ZmWRKY* genes were mapped to all 10 chromosomes of maize with an uneven distribution (Fig. 2). The chromosome 8 contained the largest number of *ZmWRKY*s (26 genes) and the second was the chromosome 3 (24 genes). The least number of *ZmWRKYs* was found on the chromosome 9, with only five genes. All chromosomes contained the members from all three groups in addition to the chromosome 5, which only had the Group II members.

**Fig. 3:**
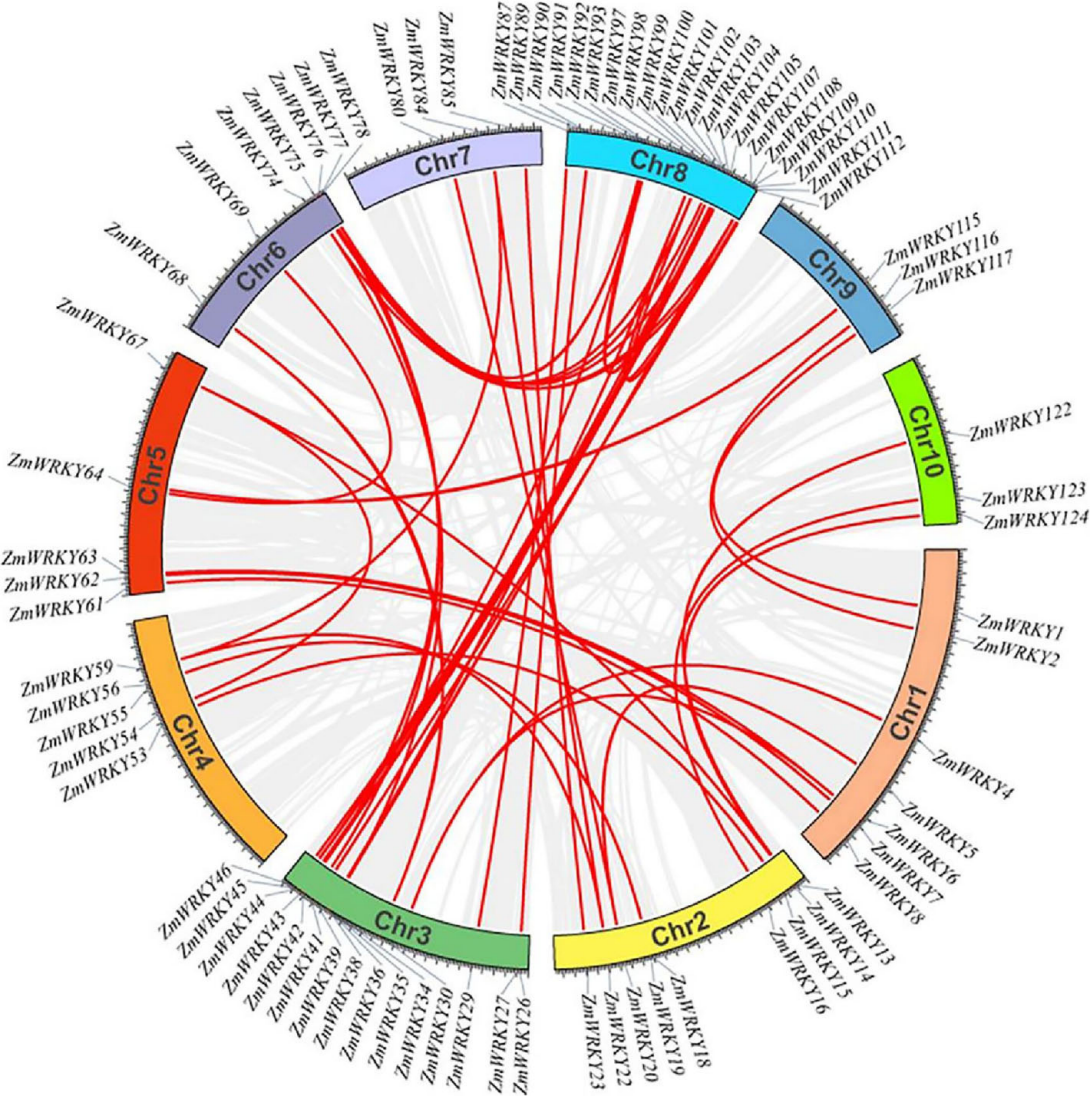
Synteny analysis of *ZmWRKY* genes in maize genome. Duplicated blocks in maize chromosomes were revealed. The circular image retrieved from PLAZA database show inter-chromosome homologous regions connected by bands in different colors. The chromosome numbers and *ZmWRKY* genes are indicated outside. Gray lines mean all syntenic regions in whole maize genome and red lines mean *ZmWRKY* gene pairs with segmental duplication

The gene duplication events were analyzed to reveal the expansion mechanism of the maize *WRKY* gene family. Among the *ZmWRKY* genes, totally 52 gene pairs were involved in gene duplication events. Holub [24] defined the tandem duplication event as a chromosome region within 200 kb including two or more genes. All the 52 *ZmWRKY* gene pairs among 78 *WRKY* genes (Table S4) were recognized as segmental duplication, but no tandem duplication was identified, indicating that tandem duplication events might have not taken part in the amplification of *ZmWRKY* gene family. Most of the duplications were located between the chromosomes 3 and 8 (Fig. 3).

To further estimate the origin and evolutionary history of the *ZmWRKY* gene family, two comparative syntenic maps were constructed among maize, rice and *Arabidopsis* at genome-wide levels, respectively. As shown in Fig. S2, we finally identified 132 orthologous gene pairs between maize and rice, but only 6 orthologous gene pairs between maize and *Arabidopsis.* More details about these orthologous gene pairs were shown in Table S5 and S6. There were far more orthologous genes between maize and rice than that between maize and *Arabidopsis*, which was probably resulted from the nearer phylogenetic relationship between maize and rice [23].

To determine the selection pressure on different duplicated *WRKY* genes, the *Ka* and *Ks* substitution rates and the *Ka*/*Ks* ratios for each repeat *ZmWRKY* gene pair were calculated, respectively. Normally, a ratio of 1 indicates neutral selection; a *Ka*/*Ks* ratio > 1 means adaptive evolution with positive selection, while a ratio < 1 means negative or purifying selection - i.e. evolutionary pressure to conserve the ancestral state [25]. As a result, all the *Ka*/*Ks* ratios for the 52 segmentally duplicated gene pairs were < 1 (Table S4), indicating that the maize *WRKY* gene family is highly conserved during evolution. According to a substitution rate of 6.5 × 10^− 9^ substitutions per synonymous site per year, the divergence time of duplicated *ZmWRKY* gene pairs was estimated to range from 9.6653 to 153.9372 Mya (Table S4).

### Gene structure and conserved motifs analysis of ZmWRKY genes

**Fig. 4:**
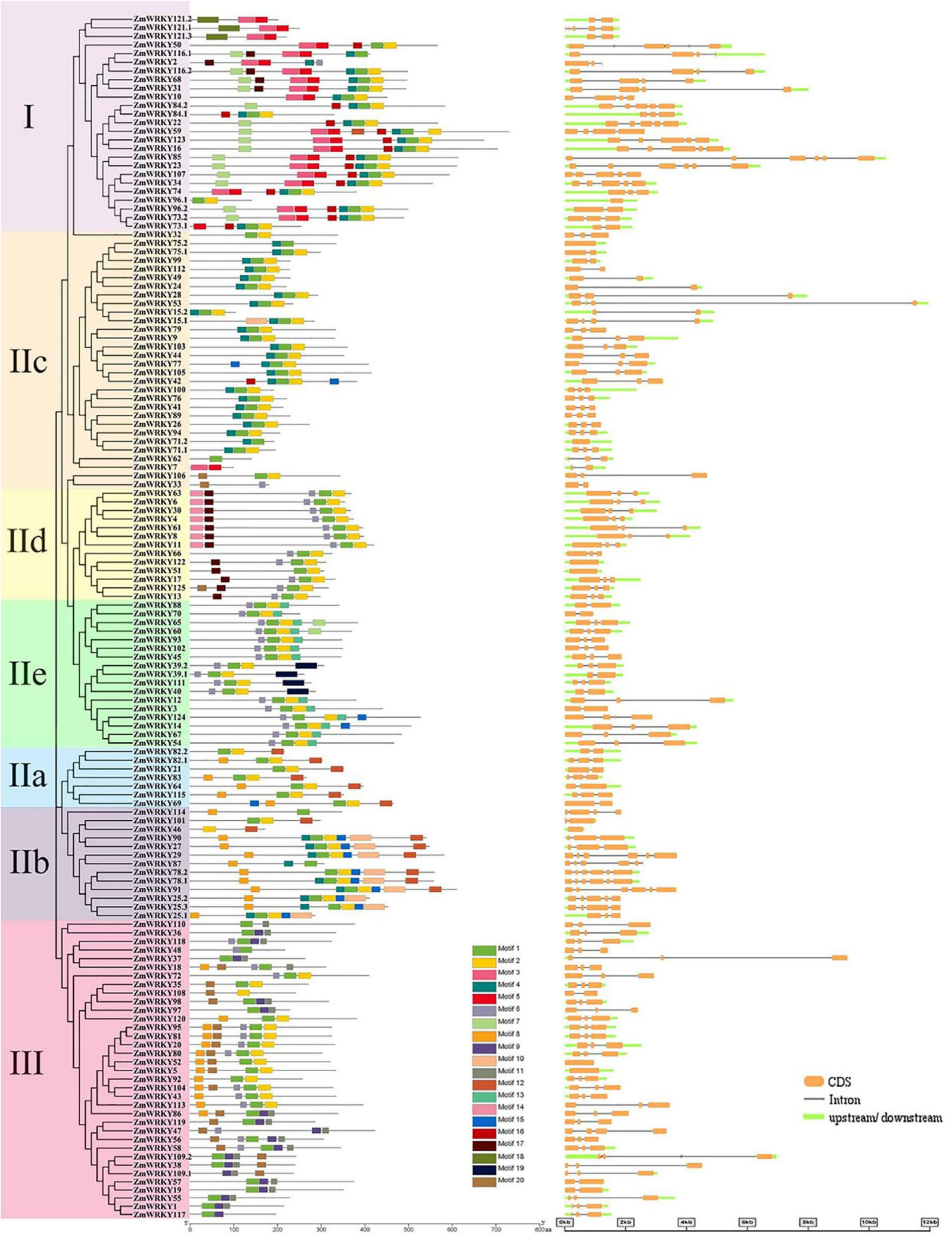
Unrooted phylogenetic tree, gene structures and conserved motifs of the ZmWRKY family in maize. The evolutionary tree from the ZmWRKY protein sequences was constructed using the MEGA 7.0 program with the Neighbor-joining method. Different colors represented various groups. The conserved motifs of ZmWRKY proteins were analyzed using MEME. Boxes with different colors denote different motifs and their position in each ZmWRKY protein. All 125 genes’ structures were obtained using GSDS 2.0. CDS, intron and 5′/3′ UTR are denoted by orange rectangles, single lines and green rectangles, respectively. (Color figure online)

The diversity of gene structure promotes the evolutionary process of the large gene families. To obtain a much more in-depth insight on evolution of the *WRKY* gene family in maize, we mapped the genetic structure of each *ZmWRKY* gene. A phylogenetic tree was constructed according to the full-length ZmWRKY proteins (Fig. 4) to better analyze gene structure and conserved motifs. The number of introns in *ZmWRKY* genes ranged from 0 to 5. The majority of *ZmWRKY* genes contained one to three introns, for 78 members containing two introns; 26 containing one intron; and 13 containing three introns. The other 8 and 7 *ZmWRKY* genes contained four and five introns, respectively. Additionally, the remaining 8 *ZmWRKY* genes contained no intron. As shown in Fig. 4, most *ZmWRKY* genes within the same group or subgroups contained a similar gene organization, indicating the functional similarity shared among different members.

To gain a better understanding of the similarity and dissimilarity of motifs in different ZmWRKY proteins, 20 conserved motifs were identified within proteins by the MEME program. MEME motif analysis exhibited that each ZmWRKY protein had its specific conserved motifs (Table S2). As depicted in Fig. 4, the WRKY family members with similar motif structures were divided into the same group. Almost all ZmWRKYs had the conserved heptapeptides WRKYGQK (Motif 1 or Motif 3), and all contained at least one motif. Moreover, the Motif 2 was comprised of the C_2_H_2_ motif, while the Motif 11 was made up of the C_2_HC motif. The conserved motif analysis demonstrated that the conserved motifs were specifically present in different groups. For instance, the Group IIa members contained 4 conserved motifs (Motifs 1, 2, 8 and 12); 13 members of the Group IIb contained 7 conserved motifs (Motifs 1, 2, 4, 8, 10, 12 and 15); the members of the Group IId contained 5 conserved motifs (Motifs 1, 2, 6, 14 and 17). It was clear that a few motifs particularly existed in one or more groups and subgroups. For instance, the Motifs 3, 5 and 16 existed in the Group I members, and the Motifs 9 and 11 existed only in the Group III, while the Motif 17 was mainly present in the Group IId. This result indicated the different roles that these groups might play in evolution and function.

### The cis-elements in the promoters of maize WRKY genes

In order to confirm the potential function of *ZmWRKY* genes in abiotic stress responses, the 2000 bp promoter sequences of the *ZmWRKY* genes were extracted and analyzed for cis-elements using the PlantCARE database. As depicted in Fig. S3, totally eleven types cis-acting elements in relation to stresses and phytohormone responses were discovered in the promoters of *ZmWRKY* genes, including four abiotic stress-related elements (W box, MBS, LTR and TC-rich repeats) and seven hormone-related elements (ERE, ABRE, GARE-motif, TCA-element, TGA-element, CGTCA-motif and TGACG-motif). As shown in Fig. S4, the CGTCA-motif and TGACG-motif (MeJA-responsive elements) were the most numerous elements in the promoter regions of 125 *ZmWRKY*s, with about 116 genes containing these two elements. ABRE (ABA-responsive elements) also appeared frequently in *ZmWRKY* gene promoter regions, which was found in 114 promoters. Several W-boxes were detected in 88 *ZmWRKY* gene promoters, suggesting that these genes have the likelihood to be regulated by other WRKY TFs or themselves. LTR and MBS elements that respond to low temperature and drought stresses were detected in the promoters of 56 and 63 *ZmWRKY* genes, respectively. Thirty promoters had a GARE-motif (gibberellin- responsive element) and only 19 promoters showed a TC-rich repeats element (cis-acting element participated in defense and stress response). Therefore, the cis-element analysis revealed that the expression of *ZmWRKY* genes in maize might be associated with different environmental factors.

### Analysis of expression profiles of maize WRKY genes in different tissues

**Fig. 5:**
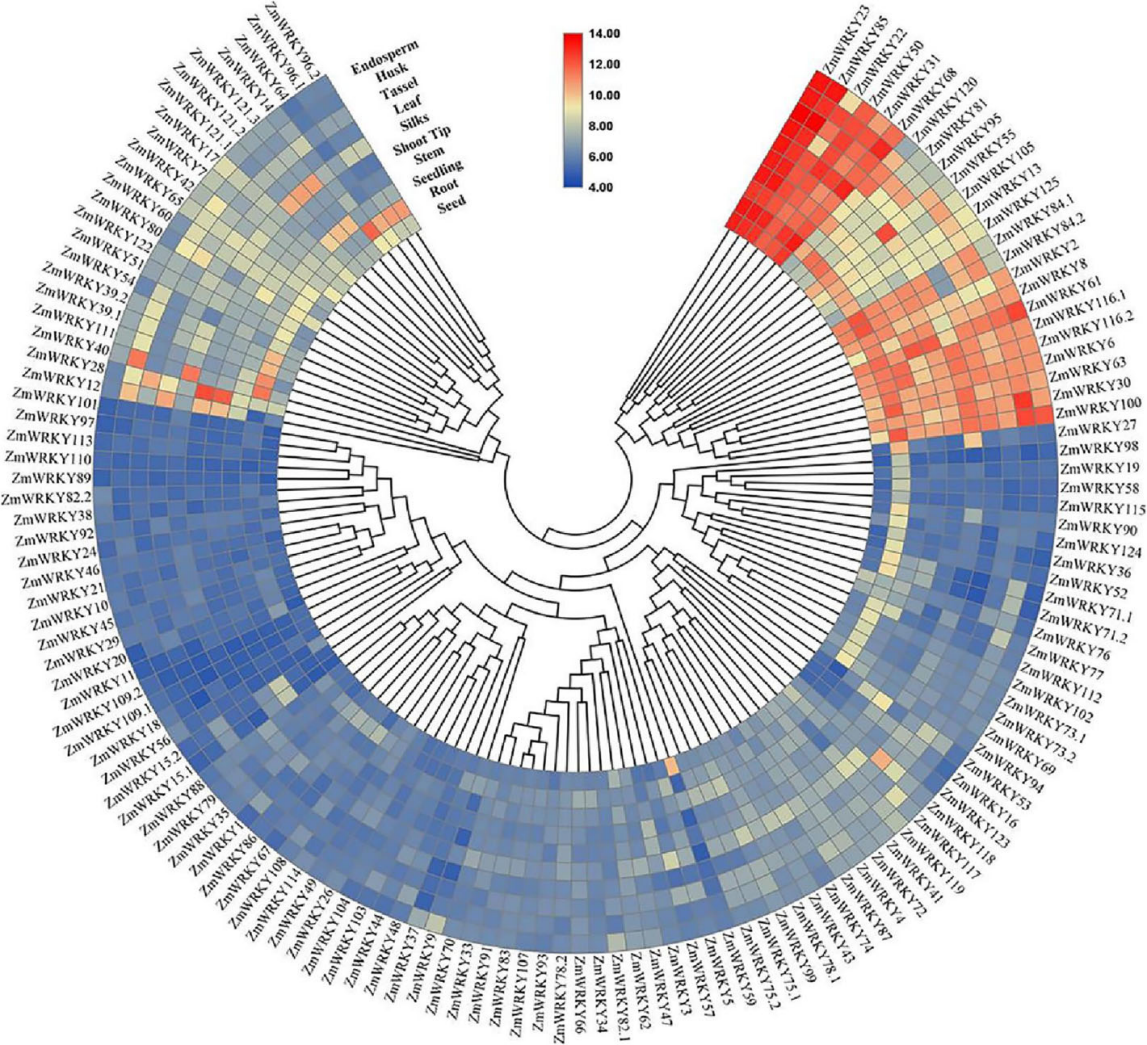
Hierarchical clustering of expression profiles of *ZmWRKY* gene family in 10 tissues. Ten tissues from different developmental stages including endosperm, husk, tassel, leaf, silks, shoot tip, stem, seedling, root and seed were investigated .The expression values were shown as log2 of the RPKM values. The scale bar is shown in the middle and higher expression levels are presented as red color

In this study, to investigate the potential functions of putative *ZmWRKY* genes in plant growth and development, the expression profiles of *ZmWRKYs* in 10 tissues at different developmental stages were analyzed according to the microarray data (Fig. 5). However, the expression data for only 135 *ZmWRKY* transcripts was revealed as shown in Table S7. The expression data of other five transcripts (*ZmWRKY25.1, − 25.2, − 25.3, − 32* and *− 106*) were not detected, indicating that these genes may be pseudogenes or have particular temporal and spatial expression patterns not explored in this database. All 135 *ZmWRKY* transcripts investigated were expressed in all tissues, although most members were only expressed at low levels, which suggested that these TFs might work with other proteins in a synergistic or interactive manner during plant growth and development. Notably, 15 *ZmWRKY* transcripts (*ZmWRKY2, − 22, − 23, − 31, − 50, − 68, − 85, − 116.1* and *− 116.2* from the Group I, *ZmWRKY6, − 8, − 30, − 61* and *− 63* from the Group IId, and *ZmWRKY100* from the Group IIc) exhibited high expression levels in all ten tissues, suggesting that these genes might play a fundamental role in the maize growth and development. What’s more, a few genes exhibited preferential expression in various tissues. For example, *ZmWRKY*30, *ZmWRKY*55, *ZmWRKY*59 and *ZmWRKY*118 showed the highest transcript abundances in husk, silks, seed and stem, respectively. In addition, several genes were discovered to be highly expressed in roots particularly. These genes might share special functions in the specific tissues. Additionally, several gene pairs with close relationship, such as *ZmWRKY*23/85, *ZmWRKY*31/68, *ZmWRKY*81/95 and *ZmWRKY16*/123, exhibited similar expression profiles, indicating that the functionality of these genes might be redundant.

### Expression profiles of maize WRKY genes under abiotic stress treatment

**Fig. 6:**
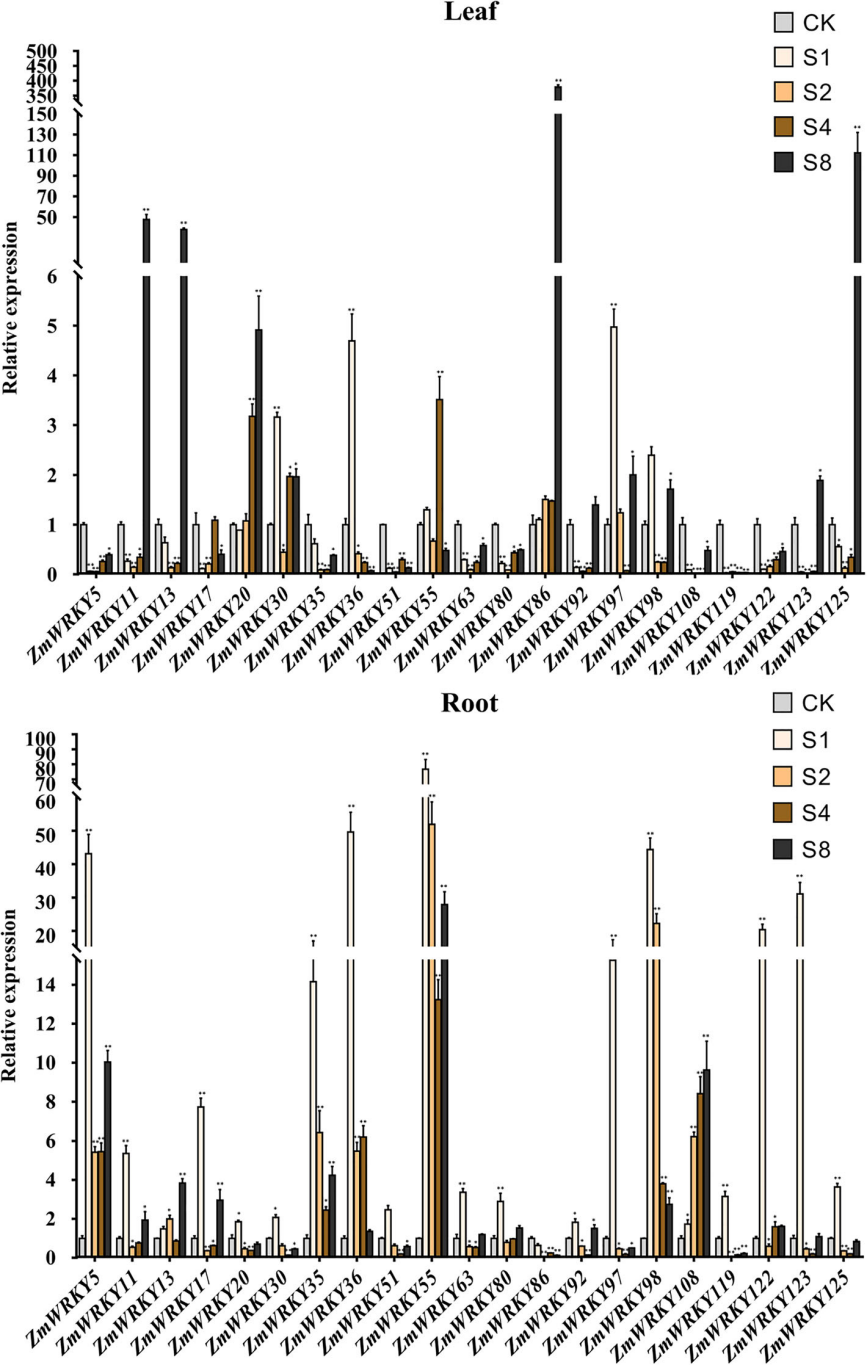
Expression profiles of 21 *ZmWRKY* genes under slat stress treatments in two different tissues. QRT-PCR data was normalized using maize Actin gene. X-axes represent various treatments (CK, normal condition; S1, S2, S4 and S8 indicate hours of salt treatment.) and different genes and y-axes are scales of relative expression level. Error bars result from three biological replicates. Asterisks on top of the error bars represent the significance levels. *Significantly different at *P* < 0.05; ** significantly different at *P* < 0.01

**Fig. 7:**
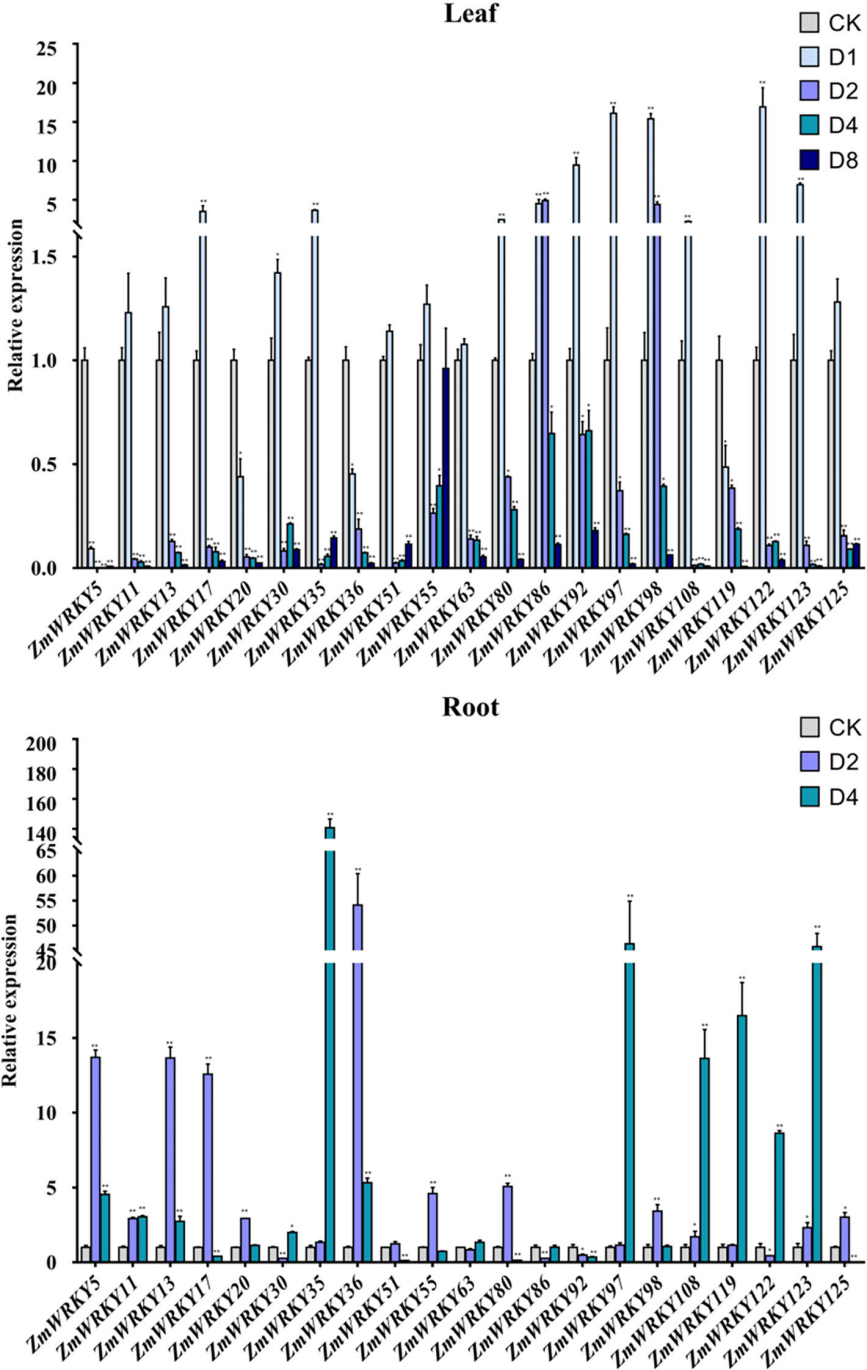
Expression profiles of 21 *ZmWRKY* genes under drought stress treatments in two different tissues. QRT-PCR data was normalized using maize *Actin* gene. X-axes represent various treatments (CK, normal condition; D1, D2, D4 and D8 indicate hours of drought treatment in leaves. D2 and D4 indicate hours of drought treatment in roots.). And different genes and y-axes are scales of relative expression level. Error bars result from three biological replicates. Asterisks on top of the error bars represent the significance levels. *Significantly different at P < 0.05; ** significantly different at P < 0.01

Under unfavorable circumstance, many stress-induced genes are in respond to help plants defend various adversity stresses. To further certify the stress-responsiveness of *ZmWRKY* genes to abiotic stresses, quantitative RT-PCR was accomplished for twenty-one *ZmWRKY* genes randomly chosen from the Group III and the Group IId, including 8 Group IId members and 13 Group III members (Figs. 6 & 7). The seedling leaves and roots were sampled at different time points to analyze their dynamic response to salt and drought stresses using qRT-PCR (Figs. 6 & 7). Under salt treatment, ten *ZmWRKY* transcripts (*ZmWRKY5, − 17, − 35, − 51, − 63, − 80, − 92, − 108, − 119* and *− 122*) didn’t show up-regulation in leaves, but they were all up-regulated in roots at different time points, while only one *ZmWRKY* transcript (*ZmWRKY86*) exhibited down-regulation in roots at different time points and most of the remaining *ZmWRKY* genes showed significant up-regulation in roots after salt treatment, indicating that these genes may play important roles in the response to salt stress. Furthermore, under salt treatment, most of these genes exhibited more rapid and much stronger responses in roots than that in leaves. For example, there were only 4 *ZmWRKY* genes (*ZmWRKY11, − 13, − 86* and *− 125*) responding to salinity and peaked at 10-fold greater levels at 8 h after salt treatment in leaves, but 8 *ZmWRKY* genes were shown to exhibit a high expression level and rapidly peaked at 14-fold greater levels at 1 h after salt treatment in roots. Among them, the transcription level of *ZmWRKY55* was up-regulated and peaked at 80-fold greater levels at 1 h. Furthermore, we further examined the responsiveness of these selected genes in response to drought stress, and obtained a similar expression pattern either under drought treatment or under salt treatment. Likewise, under drought treatment, in leaves, most genes exhibited an up-regulation in varying degrees after 1 h of drought treatment but were down-regulated with the prolonging of treatment time. Interestingly, no matter under drought treatment or salt treatment, the responses of these genes in roots were much stronger than that in leaves. For example, there were only 4 *ZmWRKY* genes (*ZmWRKY92, − 97, − 98* and *− 122*) exhibiting a high expression level (increased > 10 folds) in leaves under drought treatment, but 9 *ZmWRKY* genes were expressed highly at 10-fold greater levels in roots. The transcript level of *ZmWRKY35* even changed at 150-fold greater levels in roots. These results suggested that *ZmWRKY* genes in roots might participate in plant responses to salt stress and dehydration more strongly or rapidly than that in leaves when respond to these stress conditions.

### Weighted gene co-expression network analysis

**Fig. 8:**
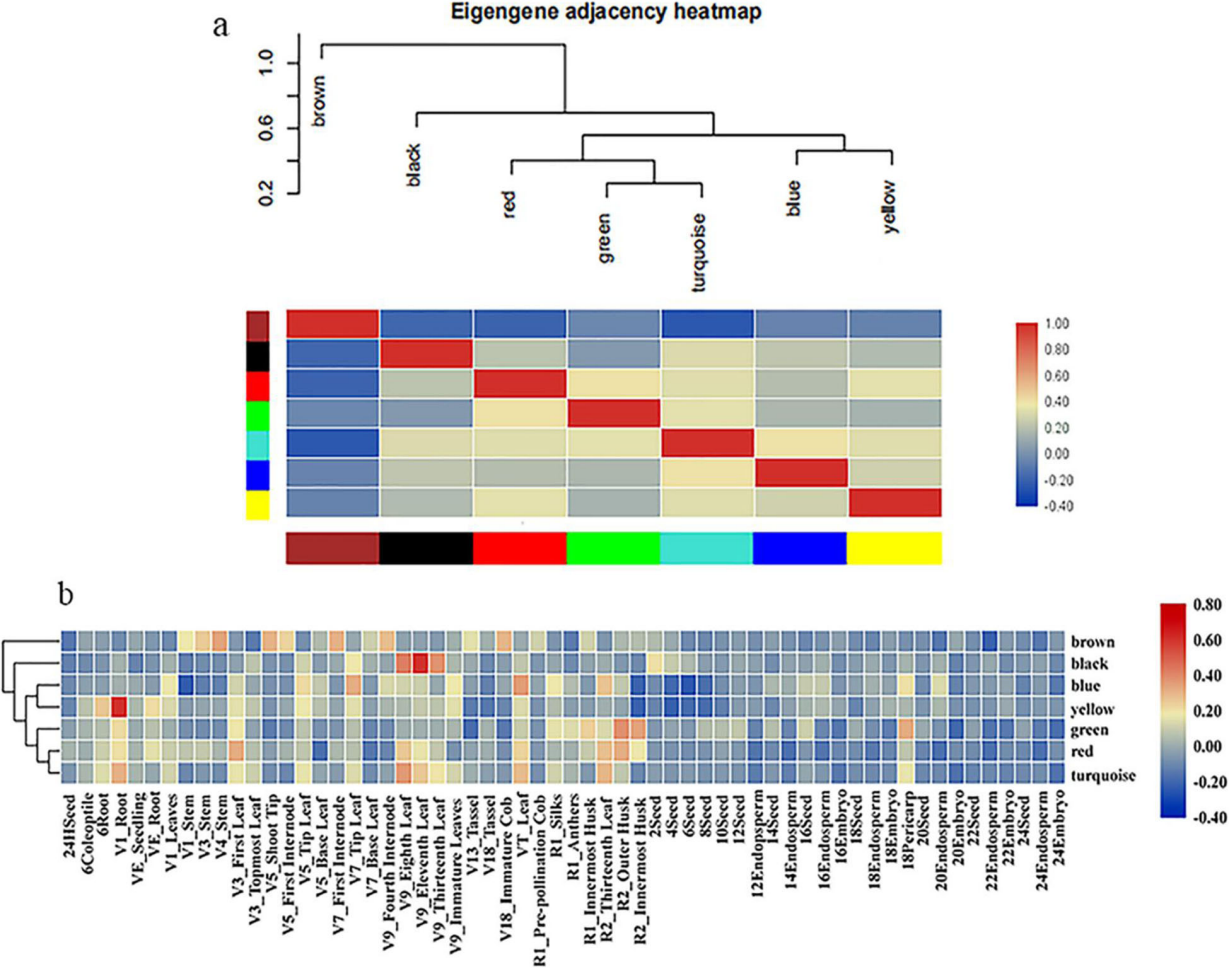
Co-expression network analysis of *ZmWRKY* genes. (**a**) Elgengene adjacency heatmap. A high degree of correlation between modules is indicated by red. (**b**) Module sample correlation. Each row corresponds to a module. Each column corresponds to specific tissue. The color of each cell at row-column intersection indicates the correlation coefficient between the module and the tissue. A high degree of correlation between a specific module and tissue type is indicated by red

To further confirm gene association patterns between different samples and identify highly coordinated genes, a matrix with 60 samples in row names 135 genes in column names was obtained from the database to perform a co-expression network analysis using Weighted Gene Co-Expression Network Analysis. The standardized sample gene expression profile matrix was used as the input file for co-expression network construction. By constructing a hierarchical clustering tree of 135 genes from 60 tissue samples in maize, it was revealed that there were no obvious outliers (Fig. S5). In order to be more accord with the scale-free features, a β value of 5 was selected to construct a co-expression network. Through the dynamic cutting tree method for module identification, a total of 7 modules were obtained (Fig. S6). The genes that could be included in any module were placed in the gray module and deleted in the subsequent analysis. The TOM heatmap was designed according to the interaction relations among the 7 modules (Fig. S7). The results showed that each module has been independently authenticated with another module, suggesting the high degree of individuality between the modules and the relative independence of gene expression in each module. In addition, the eigengenes were also calculated and clustered according to their correlation to explore the co-expression similarity of modules. We found that these 7 modules were mainly divided into three clusters (Fig. 8). The heat map drawn based on the adjacency relationship exhibited similar results.

**Fig. 9:**
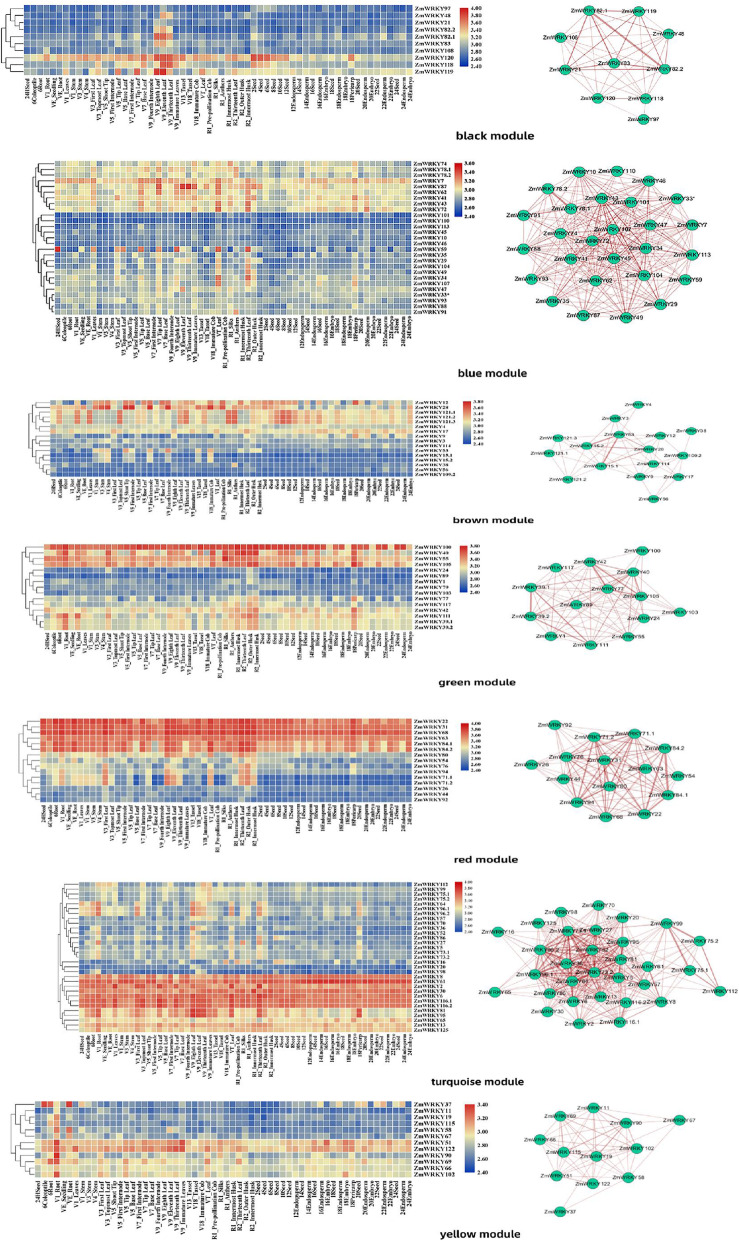
Co-expression network analysis of modules of *ZmWRKY* genes. Heatmaps showing genes in each module that were expressed in tissues. Right correlation networks in the module corresponding to heatmap. Network is visualized in Cytoscape. Green and red color Spheres (nodes) represent *ZmWRKY* genes, and the thick lines (edges) represent the high correlation

In Fig. 9, it was revealed that there were 10 genes identified in black module specific to the leaf (V9) across all the developmental stages. Of them, *ZmWRKY120* was highly expressed in most of tissues. In the network of black module, *ZmWRKY21*, *ZmWRKY82.1*, *ZmWRKY82.2* were highly related to *ZmWRKY83*. The blue module (27 genes) was associated with the leaves (VT and R1). The brown module, representing 16 genes, was highly associated with the stems (V1, V3 and V4). The network of brown module showed that *ZmWRKY53* was highly related to *ZmWRKY15.1* and *ZmWRKY15.2*. The green module, containing 15 genes, was related to the husks (R1 and R2) and periearp. In particular, three genes (*ZmWRKY55*, *ZmWRKY100* and *ZmWRKY105*) showed high expression levels in all tissues. The red module (15 genes) and turquoise module (31 genes) were highly associated with the root (V1), leaves (V3, V9, VT and R2) and husk (R2). In the network of red module, *ZmWRKY22* was highly related to *ZmWRKY84.1* and *ZmWRKY84.2*. The last module, yellow module (12 genes), was related to the roots (V1 and VE). Among them, *ZmWRKY51* and *ZmWRKY112* showed high expression levels in all tissues.

### Subcellular localization of ZmWRKY proteins

In view of the fact that subcellular location information can provide some clues for protein function research, the online software Wolf PSORT was used to predict subcellular locations of ZmWRKY proteins in this study (Table S8). The prediction results of subcellular localization indicated that most ZmWRKY proteins are mainly located in the nucleus, while there are still a few ZmWRKY proteins located in various organelles such as chloroplast, mitochondrion, cytoplasm, endoplasmic reticulum and extracellular. The results revealed that these newly-identified ZmWRKY proteins in maize exhibited a various subcellular distribution, which may be associated with functional diversification in abiotic stress responses.

### GO annotation and interaction analysis of specific ZmWRKY proteins

**Fig. 10:**
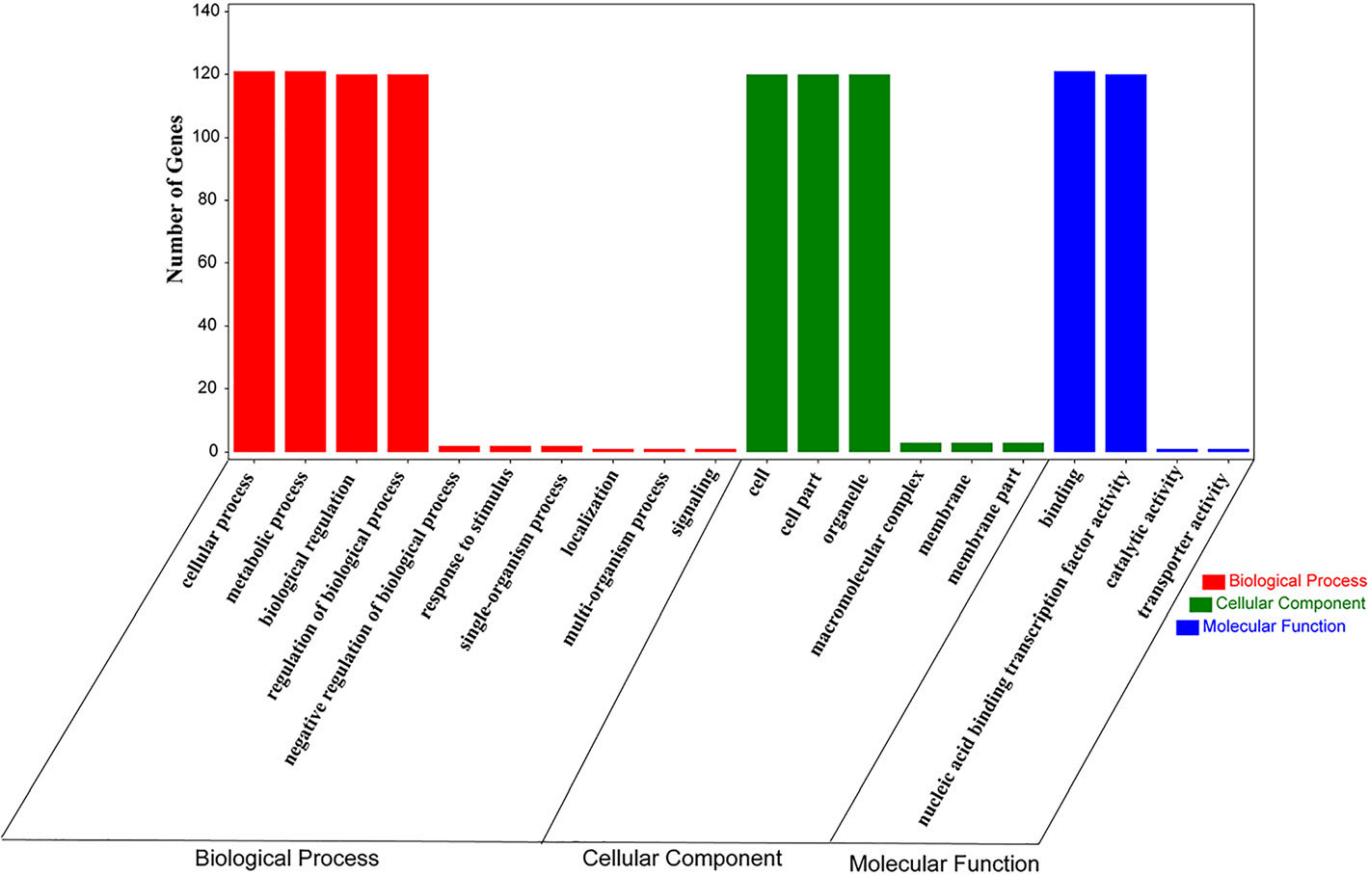
GO analysis of ZmWRKY proteins. The results are grouped into three main categories: biological process, cellular component and molecular function. The right y-axis indicates the number of genes

To explore the biological and molecular functions of ZmWRKY proteins, GO annotation and PPI analysis were further conducted in this study. The GO enrichment analysis was composed of three parts: biological process (BP), cellular component (CC), and molecular function (MF). Among the protein sequences annotated by the GO database, a total of 121 ZmWRKY proteins were divided into 3 categories and 20 subcategories (Fig. 10). In the biological process category, the proteins were distributed into 10 subcategories, and the major subcategories were ‘metabolic process’ (GO: 0008152, 121 sequences, 100%), ‘cellular process’ (GO: 0009987, 121 sequences, 100%) and ‘regulation of biological process’ (GO: 0050789, 120 sequences, 99.2%), followed by ‘biological regulation’ (GO: 0065007, 120 sequences, 99.2%). In the Cellular Component, ‘cell’ (GO: 0005623), ‘organelle’ (GO: 0043226) and ‘cell part’ (GO: 0044464), with each having 120 (99.2%) sequences. In the molecular function category, ‘organic cyclic compound binding’ (GO: 0005488, 121 sequences, 100%) and ‘nucleic acid binding transcription factor activity’ (GO: 0001071, 120 sequences, 99.2%) had the highest representation.

**Fig. 11:**
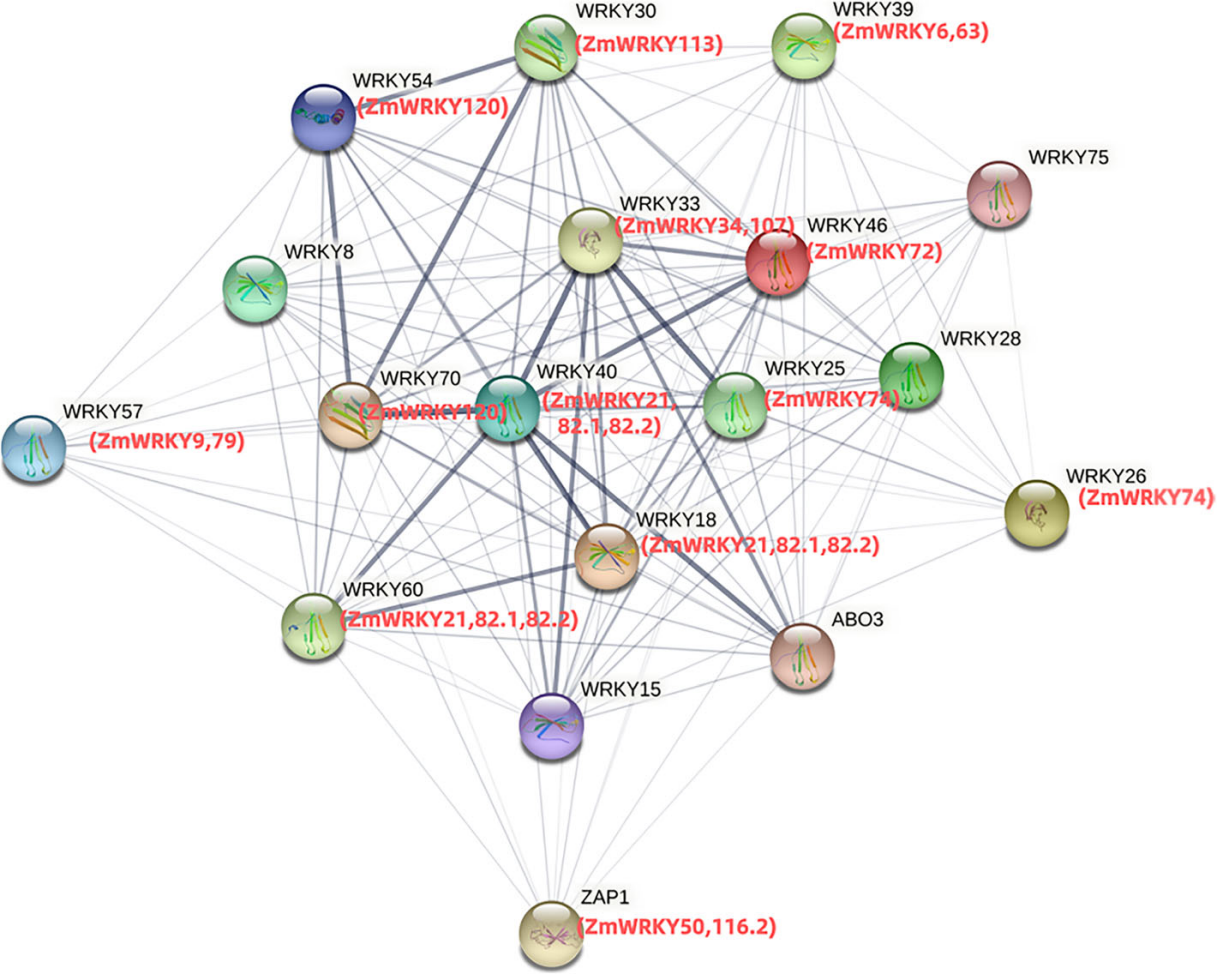
Protein Protein Interaction network of specific ZmWRKY proteins. Black and red color characters represent *Arabidopsis* and maize, and the thick lines represent the high interaction

Moreover, a protein-protein interaction network of 17 ZmWRKY proteins involved in stress responses was constructed using STRING 10.5 software based on the *Arabidopsis* association model (Fig. 11). Among the 17 ZmWRKY proteins, there were five proteins belonging to the Group I, eight proteins belonging to the Group II, and two proteins belonging to the Group III, respectively. In addition, AtWRKY40 (ZmWRKY21, ZmWRKY81.1 and ZmWRKY81.2), AtWRKY33 (ZmWRKY34 and ZmWRKY107), AtWRKY30 (ZmWRKY113), AtWRKY18 (ZmWRKY21, ZmWRKY81.1 and ZmWRKY81.2) and AtWRKY70 (ZmWRKY120) were also involved in a stronger interaction network with other proteins.

## Discussion

### Identification and characterization of ZmWRKY genes

As far as is known, the *WRKY* gene family has been revealed to take part in regulating multiple biological processes especially in responses to various environmental stresses [26, 27]. The genome-wide analyses of *WRKY* gene families have been extensively performed in lots of higher plants whose whole genome sequencing have been completed, including *Arabidopsis*, rice, pear, grape and tomato [8, 23, 27–29]. In this study, a genome-wide analysis of maize *WRKY* genes was accomplished. A total of 125 *WRKY* genes were finally identified from the latest maize genome database. What’s more, we also found that there were lots of alternative splice isoforms in *ZmWRKY* genes and their different transcripts were further analyzed including some properties listed in Table S1.

Multiple sequence alignment showed that most ZmWRKY proteins shared extremely conserved heptapeptides (WRKYGQK) at their N-terminals and a zinc-finger-like structure (C_2_H_2_ or C_2_HC type) at their C-terminals. Nonetheless, some variations, such as WKKYGQK, WRKYGEK and WRKYGKK, have also been revealed in this study. This phenomenon has also been discovered in the *Arabidopsis*, rice, grape, potato and apple WRKY members [8, 23, 28, 30, 31]. It is speculated that these variations have the possibility to change the binding specificity of DNA targets and influence the expression status of stress-responsive genes targeted by ZmWRKY TFs. In addition, it was revealed that seven ZmWRKY proteins that did not have a whole zinc finger motif and three proteins without the complete WRKYGQK sequence were also identified as the ZmWRKY family members. The gain and loss of domain might be one of the reasons for the expansion of *WRKY* gene family in maize.

In this study, an unrooted phylogenetic tree was established by multiple sequence alignment of conserved WRKY domains from these identified WRKY proteins in maize and some representatives selected from *Arabidopsis* and rice WRKY proteins. As depicted in Fig. 1, the ZmWRKY proteins from the Groups IIa and IIb were closer together, while the Group IId was closely related with the Group IIe. For the break of the Groups IIa and IIb and the break of the Groups IId and IIe were much later than the other Groups in the ancestor of terrestrial plants [10], it was supposed that the Groups IIa and IIb, and the Group IId and IIe should be merged into two new subfamily, IIa + b and IId + e, respectively [11, 32].

Previous studies showed that the analysis of gene structure and conserved motifs can offer some crucial clues to analyze the evolutionary relationship in a gene family [33]. The characterization of *ZmWRKY*s about gene structure and conserved motifs showed that gene structure and motifs were highly conserved among the members of the same group. Most *ZmWRKY* genes contained two introns, which is commonplace in other plants, such as sesame (33/71), chickpea (39/69), pear (59/103) and cassava (42/85) [29, 34–36]. Overall, the position and phase of introns in the same group presented a good similarity. For example, the Group I genes contained three to five introns, while most of the Group III genes shared two introns. Moreover, we identified 20 conserved motifs (ranging from 15 to 50 amino acid residues in length) in total among 140 ZmWRKY TFs (Table S2). As shown in Fig. 4, the conserved motifs were distributed in a phylogenetic group-specific manner for the proteins in the same group, implying that the molecular structure in the same group may be quite conserved in the process of evolution (Table S2; Fig. 4).

### Gene duplication events of ZmWRKY genes

Gene duplication, including tandem or segmental duplication, is a frequent occurrence during angiosperm evolution, which usually is regarded as a critical mechanism associated with the expansion and complexity of gene families [37]. In this study, there were 52 gene pairs in total identified to take part in segmental duplication events among the 125 *ZmWRKY* genes, but without a single tandemly-duplicated gene pair. This result implied that segmental duplication events have played a crucial part in the expansion of maize WRKY gene family.

In addition, we also investigated the orthologous *WRKY* gene pairs among maize, *Arabidopsis* and rice. *Arabidopsis* and rice are the most significant eudicot and monocot model plant species and gene functions in *Arabidopsis* and rice mostly have been adequately interpreted. As shown in Fig. S2, 95 (76%) *ZmWRKY* genes have one or two putative orthologs in rice; however, only six (4.8%) *ZmWRKY* genes have orthologs in *Arabidopsis.* The result was in accordance with the present explanation of plant phylogeny. Furthermore, the synteny analysis result suggested that the majority of *ZmWRKY* orthologs might emerge after the divergence of monocots and dicots. Furthermore, we also found that the six *ZmWRKY* genes which being synteny with *AtWRKYs* also shared relative orthologs in rice, thus it was suspected that these genes in these species might antedate the divergence of monocots and eudicots.

The analysis of *Ka* and *Ks* substitutions in duplicated genes is a useful way to research the evolution of important genes [38]. The *Ka/Ks* ratios of the 52 duplicated pairs revealed that these gene pairs seemed to have gone through strong purifying selection. Purifying selection usually selectively removes harmful alleles with the passage of time [39], suggesting that the *WRKY* gene family may play an essential role in the development and survival of the maize plant, which makes it necessary to protect and propagate its members. Maize genome has undergone twice whole genome duplication, and the previous one happened about 55–70 Mya before the divergence of Gramineae while the latter one after tetraploidization occurred about 4.8 Mya [40, 41]. The computational results of the duplication dates of the 52 paralogous pairs demonstrated that the divergence time ranged from 9.6653 to 153.9372 Mya (Table S4), suggesting that most of the segmental duplication events in the maize *WRKY* family did not happen until the divergence of the grasses later.

### Expression profiles of maize WRKY genes under abiotic stress treatment

The WRKY TFs regulate a series of biological processes and take part in lots of abiotic stress responses. It has been demonstrated that the Groups III and IId *WRKY* genes play a key role in plant development, stress response and even evolution. Transient expression researches on the Group III *WRKY* genes of *A. thaliana* revealed that these genes are components of various plant stress signaling pathway, no matter in compatible, incompatible or non-host interactions, suggesting their functional segregation [42]. The Group III *WRKY* genes were considered as the most vital group about gene family evolution and seemingly have played an important role during the adaptation and evolution of plants [10]. Additionally, a great deal of studies demonstrated that the Group IId WRKY proteins are important regulators in multiple biological processes in plants. Eleven members of the Group IId *OsWRKY* in rice showed significant change in expression levels under different abiotic (salt, drought, and cold) and biotic stresses [43]. In banana, the Group IId *WRKY* genes were abundant that control ethylene (ET)-related maturing, while the expression of 17 out of 25 Group IId genes was differently affected by ethylene [44]. In this study, the expression of twenty-one *ZmWRKY* genes randomly chosen from the Groups III and IId members of *WRKY* gene family in maize were subjected to salt and drought stress treatments from two different tissues and profiled by means of quantitative real-time PCR. The results demonstrated that most of these genes were differentially up-regulated at different time points under salt and drought stress treatments, while there still were few *ZmWRKY* genes down-regulated in two different tissues after stress treatments, suggesting that the maize *ZmWRKY* genes might have a positive or negative response to abiotic stress treatment, but their response changed with the degree of stress. However, the underlying mechanism of this phenomenon still remains to be further elucidated. Increasing evidence suggests that WRKY TFs not only participate in plant growth and development, but also show complex regulatory mechanisms and networks involved in external abiotic stresses in a time-dependent manner. Notwithstanding this, there is still one possibility that a function of WRKY TFs may vary as a consequence of environmental stress and in the process of adaptation [45]. Noticeably, in this study all these detected genes exhibited differential expression patterns in two different tissues under salt and drought stress treatments. It can be speculated that the majority of *ZmWRKYs* may play different roles in different tissues. One possible explanation might be the tissue-specific regulation. Moreover, many studies have demonstrated that *WRKY* genes are involved in responses to abiotic stresses, such as cold, drought and salt etc. in a variety of plant species [11]. In addition, through promoter analysis, many similar abiotic stress response cis-elements were revealed in the promoter regions of *WRKY* genes in various species, implying that most *WRKY* genes in plants might be involved in the transcriptional control of multiple abiotic stress responses. In this study, our analysis also uncovered many cis-elements related to drought, low temperature, salt and multiple hormone-related response elements in the *ZmWRKY* promoters, implying that most *ZmWRKY* genes were involved in the transcriptional control of defense and abiotic stress responses. The response of *ZmWRKY* genes to abiotic stresses can provide valuable clues to reveal the potential role of *WRKY* genes in maize. Many studies have revealed that *WRKY* genes were induced and exhibited up-regulated or down-regulated expression levels by drought, salt and exogenous hormones in a time-dependent manner, indicating that WRKY transcription factors were involved in the response to abiotic stresses through stress-related transcriptional regulatory elements. In this study, most *ZmWRKY* genes can be induced by diverse stress treatments such as salt and drought, implying that a single *WRKY* gene can be regulated by various abiotic stress responses through different cis-elements in the promoter region. Previous studies have demonstrated that the analysis of *ZmWRKY65* promoter sequence in maize indicated that it contains several stress-related transcriptional regulatory elements including ABA-responsive element, MYB-responsive element, MYC-responsive element, dehydration-responsive element and low temperature-responsive element recognition site sequences. Many environmental factors can induce the transcription of *ZmWRKY65* gene, such as drought, salinity, high temperature and low temperature stresses. Moreover, the transcription of *ZmWRKY65* gene was also affected by the induction of defense related plant hormones such as SA and exogenous ABA [46]. Furthermore, another two maize genes *ZmWRKY40* and *ZmWRKY106* were also identified to enhance the tolerances to drought and high-temperature [47, 48]. The gene and promoter structure analyses showed that there was the conservative sequence and stress-related transcriptional regulatory elements between these genes*.* Taken together, the regulatory mechanism of *WRKY* genes in responding to different abiotic stresses is complicated, and the analysis of *ZmWRKY* gene expression profiles will offer new insights into figuring out signaling pathways in maize abiotic stress responses.

### Co-expression and protein protein interaction networks analysis

Weighted Gene Co-Expression Network Analysis (WGCNA) is usually used to analyze gene function and their connection from the overall biological function by identifying functionally related or similar gene components in high-throughput data. By using the method, researchers can discover the connections among the highly coordinated genes from different modules. The modules were defined as the clusters of highly interconnected genes, and the genes within the same cluster have high correlation coefficients among them. In this study, WGCNA was used to analyze gene expression profiles of maize WRKY genes from 60 diverse tissue samples in the database, and finally seven co-expression modules were constructed as shown in Fig. 9. In the black module, *ZmWRKY21*, *ZmWRKY82.1*, *ZmWRKY82.2* and *ZmWRKY83* had a certain relationship with each other. In addition, *ZmWRKY21*, *ZmWRKY82.1* and *ZmWRKY82.2* genes are orthologs of *AtWRKY18*, *AtWRKY40* and *AtWRKY60* genes in *A. thaliana*, respectively*.* Studies have shown that *AtWRKY18*, *AtWRKY40* and *AtWRKY60* worked in cluster, and were involved in the transcriptional regulation of ABFs/AREBs by binding to the W-box element present in the promoter [49]. The maize WRKY genes, which are similar to *Arabidopsis* orthologs, were revealed that they might have similar functions in the transcriptional regulation of ABFs/AREBs. The blue module (27 genes) was associated with the leaves (VT and R1), including *ZmWRKY74* (the ortholog of *AtWRKY25* and *AtWRKY26*) and *ZmWRKY107* (the ortholog of *AtWRKY33*). It has been reported that the cross-regulation between *AtWRKY25*, *AtWRKY26* and *AtWRKY33* was necessary to promote the heat tolerance of plants [50]. Thus, it was speculated that along with them *ZmWRKY74* and *ZmWRKY107* might act as transcriptional regulators in maize response to heat stress. A growing study has demonstrated that the plant *WRKY* genes was involved in diverse biotic/abiotic stress responses as well as in developmental/physiological processes [11]. In the brown module, it included *ZmWRKY109.2* (the ortholog of *AtWRKY53*) and *ZmWRKY9* (the ortholog of *AtWRKY57*). Studies have shown that *AtWRKY53* and *AtWRKY57* were involved in regulating leaf senescence [51, 52]. In the process of JA-induced leaf senescence, it has been demonstrated that *AtWRKY57* played an important role in leaf senescence as the convergence point of JA signaling pathway and auxin crosstalk. Moreover, this module also included *ZmWRKY15.1* (the ortholog of *AtWRKY12*), *ZmWRKY15.2* (the ortholog of *AtWRKY12*) and *ZmWRKY28* (the ortholog of *AtWRKY13*). Studies have shown that *AtWRKY12* and *AtWRKY13* were involved in regulating flowering time [53]. Overall, these putative *ZmWRKY* genes, which might be correlated with transcriptional regulation of maize development, were identified in the present study by constructing a co-expression network to further infer the potential roles for these *ZmWRKY* genes and verify the function of these candidate genes associated with plant development in future.

Furthermore, based on the functional annotation and interaction analysis of WRKY proteins from other species, the possible regulatory effects of ZmWRKY proteins can be predicted. Five ZmWRKYs (ZmWRKY34, ZmWRKY107, ZmWRKY21, ZmWRKY82.1 and ZmWRKY82.2) with high sequence similarity with AtWRKY33 and AtWRKY40 were set as the central nodes of the interaction network. These five proteins were predicted to interact with other proteins according to varying degrees. The amino acid sequences of ZmWRKY21, ZmWRKY82.1 and ZmWRKY82.2 were similar to that of AtWRKY18/AtWRKY40/AtWRKY60, respectively. In *Arabidopsis*, AtWRKY18, AtWRKY40 and AtWRKY60 form homodimers or heterodimers to change the resistance to pathogens [54]. Furthermore, AtWRKY25 (ZmWRKY74), AtWRKY26 (ZmWRKY74) and AtWRKY33 (ZmWRKY107) actively regulate the synergy between ethylene activation and heat shock protein-related signaling pathways, which mediate the response to heat stress. These three proteins interact functionally and play a synergistic role in plant heat tolerance [50]. It is generally acknowledged that homologous proteins with similar domains and sequences among different species may share the same or similar functions. Overall, the results showed that diverse interactions among ZmWRKY proteins, which were similar to the results of co-expression network analysis, demonstrating the co-expression of ZmWRKY proteins in response to multiple biotic and abiotic stresses.

## Conclusions

In conclusion, we identified 140 WRKY TFs altogether in maize. The classification, evolutionary characteristics, conserved domain and gene structure of the *WRKY* gene family in maize, together with stress-responsive cis-elements in the promoters of 125 *ZmWRKY* genes were investigated. The analyses of expression profiles based on RNA-seq method revealed their probable functions in different tissues. What’s more, twenty-one *ZmWRKY* genes were activated by salt and drought stresses, implying their potential roles in abiotic stress responses of *Zea mays L.* Finally, via analyzing the expression patterns of *ZmWRKY* genes in diverse tissue types and construction of co-expression networks of *ZmWRKY* genes, subcellular localization prediction, GO annotation and PPI analysis of ZmWRKY proteins, a comprehensive overview has been provided for further exploring the function and regulatory mechanism of *ZmWRKY* genes in maize, which will help in elucidating their exact function in maize.

## Materials and methods

### Plant materials, abiotic treatment, and tissue collection

The maize seeds in this experiment were provided from National Engineering Laboratory of Crop Stress Resistance Breeding, Anhui Agricultural University. Maize (*Zea mays L.* inbred line B73) plants were cultivated in an artificial climate chamber at 28 °C with long-day conditions of 14 h of light and 10 h of dark and an environmental humidity of 50%. To impose the drought treatment, we gently pulled the whole maize seedlings out of the soil. For the salt-stress treatment, the maize seedling roots were submerged in a 200 mM NaCl solution. Seedlings maintained with enough water at 28 °C in the dark were used as controls. The seedlings were collected at 1, 2, 4, and 8 h after the drought or salt treatment. Then the samples of seedling roots and leaves were rapidly frozen in liquid nitrogen, and immediately stored at − 80 °C until RNA extraction.

### Identification of WRKY genes in maize

The latest DNA and protein sequence information of B73 maize were obtained from phytozome 12.1 (http://www.phytozome.net) [55]. A similar approach was used to identify the maize WRKY proteins just as reported in other plants [56]. The Hidden Markov Model (HMM) profile for the WRKY domain from the Pfam database (http://pfam.janelia.org) was used to identify WRKY proteins from the maize genome. Later, the Pfam database was used to check if all candidates contained the WRKY domain. In the end, the overlapping and deficient sequences were excluded through manual inspection in MEGA 7.0 [57]. The basic information of maize WRKY TFs such as the protein sequence length, open reading frame (ORF) length and chromosome location was got from the online project MaizeGDB (https://chinese.maizegdb.org). The molecular weight (kDa) and isoelectric point (PI) of each WRKY protein were estimated by the online ExPASy Bioinformatics Resource Portal (https://web.expasy.org/compute_pi/) [58] and parameter was set as default.

### Sequence alignment and phylogenetic analysis

The conserved WRKY domain sequences of ZmWRKY proteins were aligned by DNAMAN 7.0 software. Moreover, the conserved domains were investigated by online tool WebLogo (http://weblogo.berkeley.edu). The phylogenetic tree was constructed through the neighbor-joining (NJ) method and a bootstrap value of 1000 by the MEGA 7.0 software [57]. To get a more reliable and credible result of classification of the different groups, 36 selected WRKY domains from rice and *Arabidopsis* were covered during phylogenetic analysis. Furthermore, an online program, Interactive Tree of Life (iTOL) (http://itol.embl.de/), was employed to give the phylogenetic tree a good looking.

### Chromosomal mapping, gene duplication and synteny analysis

The chromosome mapping of *ZmWRKY* genes was accomplished using MG2C (http://mg2c.iask.in/mg2c_v2.0/) based on their starting and ending positions in maize chromosomes. MCScanX was applied to identify tandem and segmental duplications of *ZmWRKY* genes [59] and the results of duplication genes were displayed by TBtools [60]. Each orthologous gene pair was then further investigated with PAL2NAL (http://www.bork.embl.de/pal2nal/) [61] to calculate the Ks (synonymous substitution rate) and the Ka (non-synonymous substitution rate). The values of Ks were further used to calculate the divergence time (T) by applying a formula T = Ks/2λ × 10^− 6^ Mya, supposing a rate (λ) of 6.5 × 10^− 9^ substitutions per synonymous site per year for maize [62]. MCScanX was also employed to carry out synteny analysis of *ZmWRKY* genes between *Arabidopsis* and rice.

### Analysis of conserved motif distribution and gene structure

The MEME Suite version 5.0.5 (http://meme.nbcr.net/meme/) was applied to analyze conserved motifs for each ZmWRKY TF. The parameters for motif identification were set as the following: maximum number, 20; number of repetitions, any; the width of each motif, ranged from 6 to 100. By the use of the online program GSDS (http://gsds.cbi.pku.edu.cn/index.php) [33], gene structure diagrams were constructed utilizing the relevant nucleotide sequences of *ZmWRKY* genes gained from phytozome 12.1 (http://www.phytozome. net) [55].

### Analysis of the cis-regulatory elements in ZmWRKY genes

To survey the *cis*-acting factors of the promoter sequence, the online tool PlantCARE (http://bioinformatics.psb.ugent.be/webtools/plantcare/html/) [63] was applied to explore the genomic sequence in 2 kb upstream region of the initiation codon (ATG) in each *ZmWRKY* gene.

### Expression profile analysis of maize WRKY genes

The standardized data (Reads/kb/Million, RPKM) for different tissues from different growth stages was provided by Sekhon et al. [64], and downloaded from MaizeGDB (http://www.maizegdb.org). Finally, we identified expression patterns of *ZmWRKY* genes in ten different tissues, including seed, root, seedling, stem, shoot tip, silks, leaf, tassel, husk and endosperm. The expression values were calculated by log2 (FPKM) and were displayed as a heat map by TBtools software [60].

### Analysis of maize WRKY gene expression under salt and drought stresses by qRT-PCR

Total RNAs were extracted from different seedling samples using RNAiso Plus (TaKaRa, Japan) in accordance with the specification. Then the RNAs were reverse transcribed into the first-strand cDNA by FastKing RT Kit (With gDNase) (TIANGEN, China). To carry on the expression profile analysis of *ZmWRKY* genes under salt or drought stress treatment, several *WRKY* genes from group III and IId were selected for research by Real-time quantitative PCR (qPCR). Three replicates were conducted on a CFX96 Touch™ Real-Time PCR detection system (BIO-RAD, USA). The maize actin gene (Accession No.: NC_024466) was used as a reference for normalization. PCR reactions used iTaq™ Universal SYBR® Green Supermix (BIO-RAD, USA). Amplification conditions were as follows: initial denaturation of 95 °C for 30 s and 40 cycles of denaturation at 95 °C for 5 s, 55 °C annealing for 20 s and extending at 72 °C for 30 s, in the end, with a melting curve to check the amplification specificity. The relative mRNA expression level for each gene was calculated as 2^-ΔΔCT^ method [65]. Gene-specific DNA primers for qPCR are presented in Supplementary Table 3. In the end, the mean ΔCt values were statistically analyzed using the Student’s t-test (http://www.physics.csbsju.edu/stats/t-test _bulk_form.html) to identify the expression profiles showing significant differences. *P* ≤ 0.05 was regarded as statistically significant.

### Weighted gene co-expression network construction

The standardized data (Reads/kb/Million, RPKM) for different tissues from different growth stages were provided by Sekhon et al. [64], and downloaded from MaizeGDB (http://www.maizegdb.org). These data include 60 samples and more than 70,000 genes. The sample data of *ZmWRKY* genes were selected for subsequent analysis. R statistical software (version 4.0) and WGCNA package were used for statistical calculations [66]. By choosing an appropriate weighting coefficient β (soft threshold), the connection between genes in the constructed network was subject to scale-free network distribution, and the correlation coefficient between genes was used to construct a hierarchical clustering tree. Different branches of the clustering tree were exhibited to represent different gene modules with different colors. Then, based on the weighted correlation coefficients of genes, the genes were classified according to their expression patterns, and the genes with similar patterns were grouped into one module, so that the genes were classified into different modules by gene expression patterns for further analysis. Finally, the coefficient was used to convert the similarity matrix into an adjacency matrix, and further into a topological overlap matrix (TOM), all genes were used as a TOM heap map to prove the high degree of independence between modules and the expression of genes in each module relative independence. Connectivity was defined as the sum of the weights across all the edges of a node, and the co-expression network was built using Cytoscape software.

### Subcellular localization of ZmWRKY proteins

The online software WOLF PSORT (https://wolfpsort.hgc.jp/) was utilized to predict subcellular localization by uploading protein sequences.

### Gene ontology annotation and protein protein interaction analysis

The GO number of the maize WRKY genes was obtained from Ensemble Plant (http://plants.ensembl.org/index.html). The hierarchical structure file was loaded in Gene Ontology website (http://geneontology.org/). Furthermore, an online tool, OmicShare (https://www.omicshare.com/tools/), was used for plotting Gene Ontology annotation results. A functional protein association network was constructed in the STRING program based on the *Arabidopsis* association model with the confidence parameter for 0.15 and the number of interactions for 5, respectively.

## Supplementary Information

**Additional file 1.**

## Abbreviations

ABA: Abscisic acid
qRT-PCR: Quantitative RT-PCR
WGCNA: Weighted gene coexpression network analysis
GO: Gene Ontology
PPI: Protein-protein interaction
ET: Ethylene
JA: Jasmonic acid
HMM: Hidden Markov Model
NJ: Neighbor-joining
Ks: synonymous substitution rate
Ka: non-synonymous substitution rate
