# Supplementary material for: Genome-wide identification and analysis of WRKY gene family in maize provide insights into regulatory network in response to abiotic stresses
Source: BMC Plant Biol. 2021 Sep 20;21:427. doi: 10.1186/s12870-021-03206-z (PMC8451115; doi:10.1186/s12870-021-03206-z)
Supplement: Supplementary file 1 — Additional file 1. [file 12870_2021_3206_MOESM1_ESM.docx]

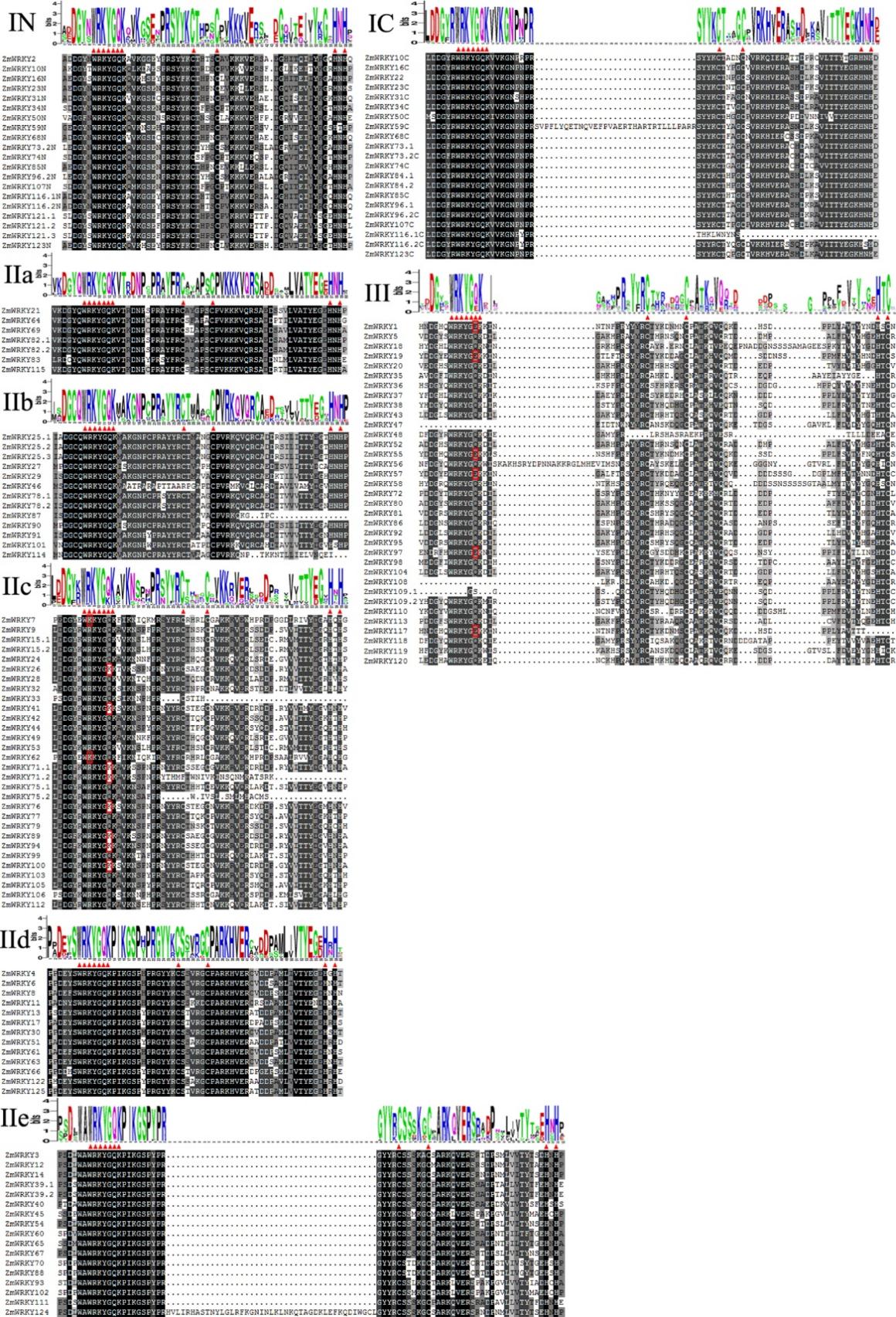


**Figure S1 Multiple sequence alignment of the WRKY domains in maize**. Alignment was using software DNAMAN. Black, dark gray, medium gray and light gray represent 100, 75, 50 and 30% similarity, respectively. The red triangles represent the highly conserved WRKYGQK and the zinc-finger motif. Variations of the heptapeptide are marked with red boxes. The conserved domains of the ZmWRKY protein sequences were analyzed using WebLogo. The Y axis (measured in bits) depicts the overall height of the stack, indicating the sequence conservation at that position, while the height of symbols within the stack indicates the relative frequency of each amino at that position. (Color figure online)


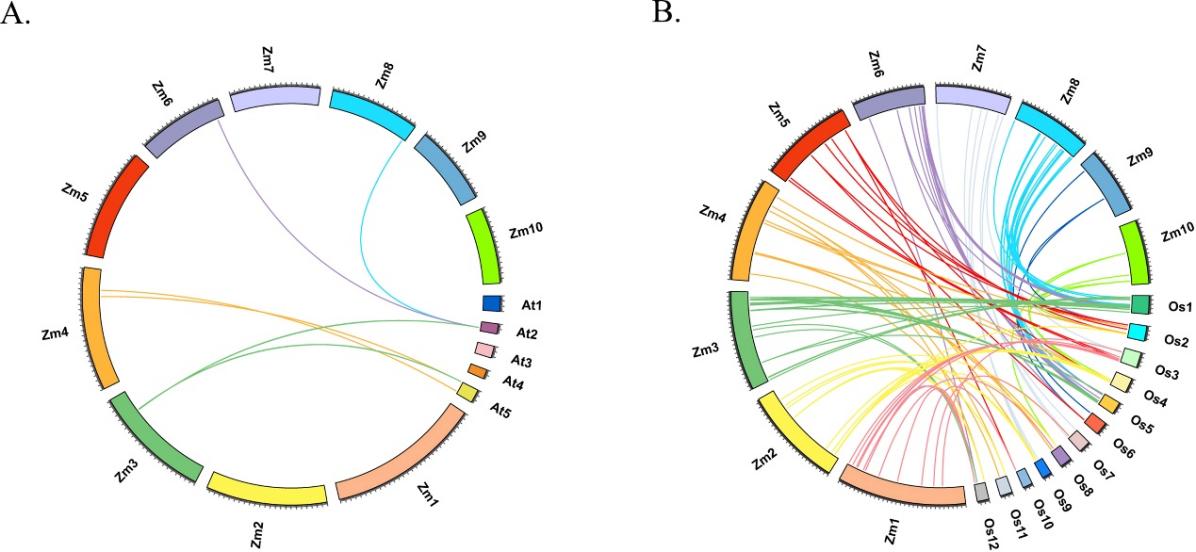


**Figure S2 Synteny analysis of *ZmWRKY* genes with the genomes of *Arabidopsis* and rice. (A)** Synteny analysis between *Zea mays L.* (Zm) and *Arabidopsis* (At). (B) Synteny analysis between *Zea mays* (Zm) and rice (Os). There were 6 and 95 *ZmWRKY* genes identified to be orthologous to *Arabidopsis* and rice, respectively.


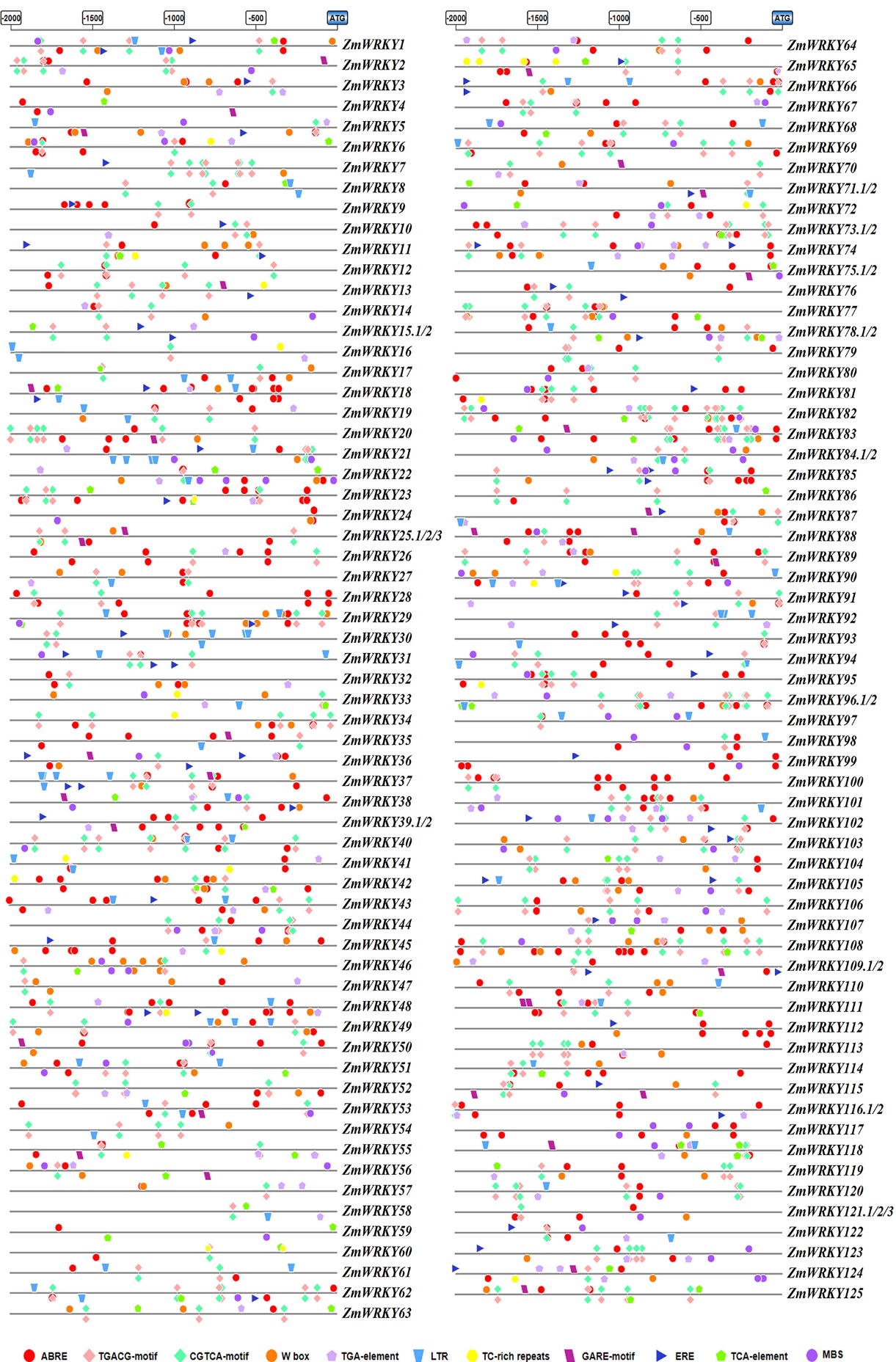


**Figure S3 Distribution of major stress-related cis-elements in the promoter sequences of the 125 *ZmWRKY* genes.** Putative ABRE, LTR, MBS, TGACG-motif, TC-rich repeats, CGTCA-motif, GARE-motif, W box, ERE, TGA-element and TCA-element core sequences are represented by different symbols as indicated in figure key at the bottom. The cis-elements distributed on the sense strand and reverse strand are indicated above and below the black lines, respectively. ABRE: cis-acting element involved in the abscisic acid responsiveness; LTR: cis-acting element involved in low-temperature responsiveness; MBS: MYB binding site involved in drought-inducibility; TGACG-motif: cis-acting regulatory element involved in the MeJA-responsiveness; TC-rich repeats: cis-acting element involved in defense and stress responsiveness; CGTCA-motif: cis-acting regulatory element involved in the MeJA-responsiveness; GARE-motif: gibberellin-responsive element; W box: elicitation; wounding and pathogen responsiveness; ERE: ethylene-responsive element; TGA-element: auxin-responsive element; TCA-element: cis-acting element involved in salicylic acid responsiveness. The 2000bp sequences upstream of the initiation codon (ATG) of the WRKY genes can be estimated using the scale per 500bp at the above.


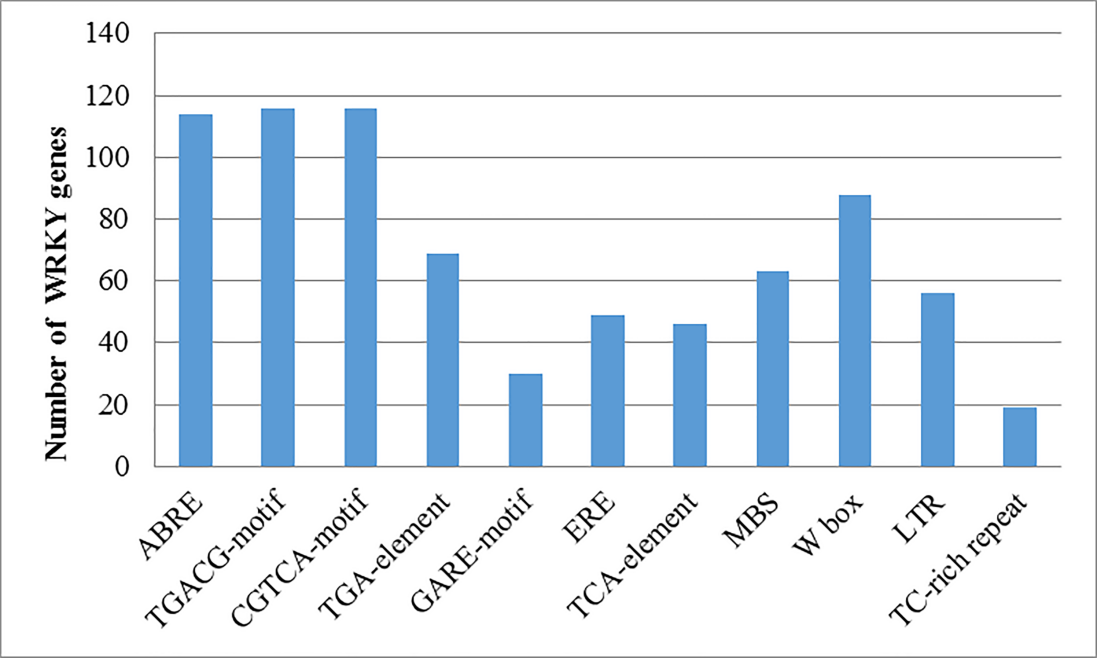


**Figure S4 The number of *WRKY* genes containing various cis-acting elements. Graph was plotted on the basis of presence of cis-regulatory element (x-axis) in *WRKY* gene family members (y-axis).**


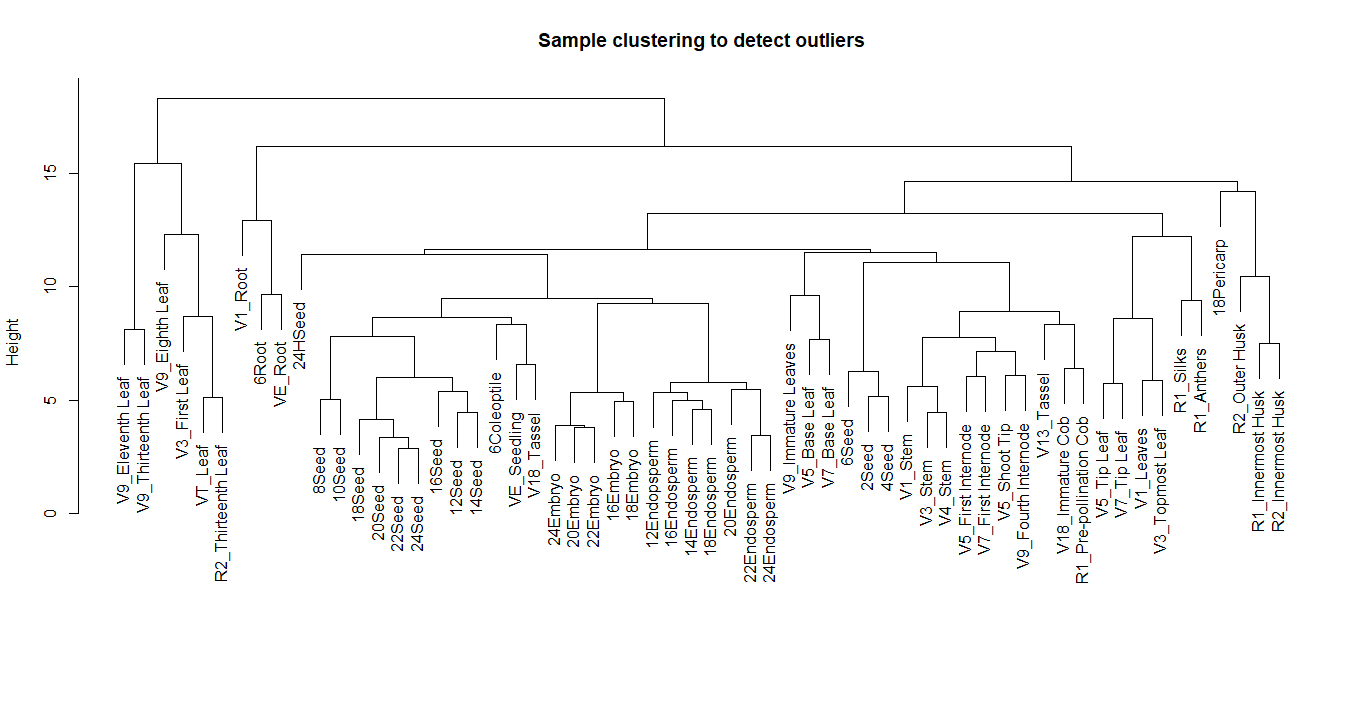


**Figure S5 Sample clustering. 60 tissue samples at different growth stages.**


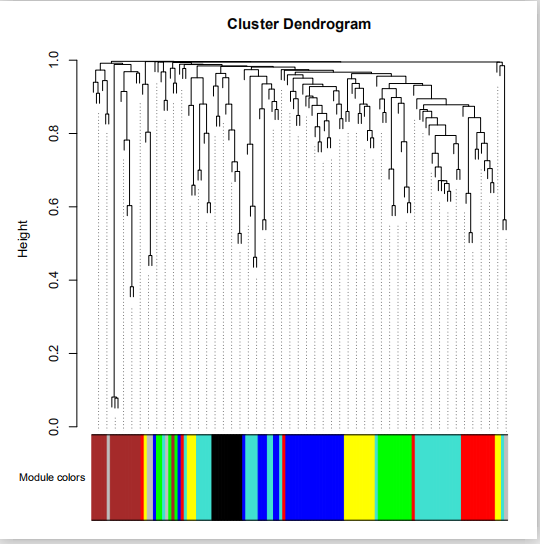


**Figure S6 Cluster Dendrogram. Different colors represent different modules. Seven modules: brown, red, yellow, blue, green, turquoise and black.**


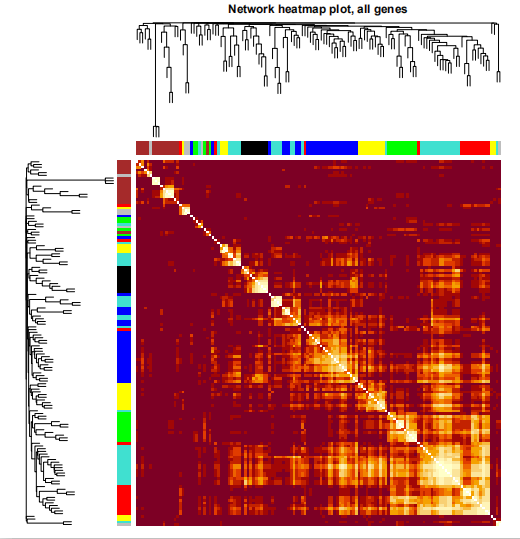


**Figure S7 Topological Overlap Matrix (TOM) heatmap.**

**Table S1** **Basic information of *WRKY* genes identified in maize**

| **Serial**  **NO.** | **Gene Name** | **Accession Number**  **Ensemble transcript** |  | **Genome Location Coordinates (5'-3')** | **ORF**  **Length**  **(bp)** | **Length**  **(aa.)** | **Protein**  **Mol. Wt.**  **(Da)** | **PI** | **Chr.** | **Type** |
| --- | --- | --- | --- | --- | --- | --- | --- | --- | --- | --- |
| **1**  **2**  **3**  **4**  **5**  **6**  **7**  **8**  **9**  **10**  **11**  **12**  **13**  **14**  **15**  **16**  **17**  **18**  **19**  **20**  **21**  **22**  **23**  **24**  **25**  **26**  **27**  **28**  **29**  **30**  **31**  **32**  **33**  **34**  **35**  **36**  **37**  **38**  **39**  **40**  **41**  **42**  **43**  **44**  **45**  **46**  **47**  **48**  **49**  **50**  **51**  **52**  **53**  **54**  **55**  **56**  **57**  **58**  **59**  **60**  **61**  **62**  **63**  **64**  **65**  **66**  **67**  **68**  **69**  **70**  **71**  **72**  **73**  **74**  **75**  **76**  **77**  **78**  **79**  **80**  **81**  **82**  **83**  **84**  **85**  **86**  **87**  **88**  **89**  **90**  **91**  **92**  **93**  **94**  **95**  **96**  **97**  **98**  **99**  **100**  **101**  **102**  **103**  **104**  **105**  **106**  **107**  **108**  **109**  **110**  **111**  **112**  **113**  **114**  **115**  **116**  **117**  **118**  **119**  **120**  **121**  **122**  **123**  **124**  **125**  **126**  **127**  **128**  **129**  **130**  **131**  **132**  **133**  **134**  **135**  **136**  **137**  **138**  **139**  **140** | ZmWRKY1  ZmWRKY2  ZmWRKY3  ZmWRKY4  ZmWRKY5  ZmWRKY6  ZmWRKY7  ZmWRKY8  ZmWRKY9  ZmWRKY10  ZmWRKY11  ZmWRKY12  ZmWRKY13  ZmWRKY14  ZmWRKY15.1  ZmWRKY15.2  ZmWRKY16  ZmWRKY17  ZmWRKY18  ZmWRKY19  ZmWRKY20  ZmWRKY21  ZmWRKY22  ZmWRKY23  ZmWRKY24  ZmWRKY25.1  ZmWRKY25.2  ZmWRKY25.3  ZmWRKY26  ZmWRKY27  ZmWRKY28  ZmWRKY29  ZmWRKY30  ZmWRKY31  ZmWRKY32  ZmWRKY33*  ZmWRKY34  ZmWRKY35  ZmWRKY36  ZmWRKY37  ZmWRKY38  ZmWRKY39.1  ZmWRKY39.2  ZmWRKY40  ZmWRKY41  ZmWRKY42  ZmWRKY43  ZmWRKY44  ZmWRKY45  ZmWRKY46  ZmWRKY47  ZmWRKY48  ZmWRKY49  ZmWRKY50  ZmWRKY51  ZmWRKY52  ZmWRKY53  ZmWRKY54  ZmWRKY55  ZmWRKY56  ZmWRKY57  ZmWRKY58  ZmWRKY59  ZmWRKY60  ZmWRKY61  ZmWRKY62  ZmWRKY63  ZmWRKY64  ZmWRKY65  ZmWRKY66  ZmWRKY67  ZmWRKY68  ZmWRKY69  ZmWRKY70  ZmWRKY71.1  ZmWRKY71.2  ZmWRKY72  ZmWRKY73.1  ZmWRKY73.2  ZmWRKY74  ZmWRKY75.1  ZmWRKY75.2  ZmWRKY76  ZmWRKY77  ZmWRKY78.1  ZmWRKY78.2  ZmWRKY79  ZmWRKY80  ZmWRKY81  ZmWRKY82.1  ZmWRKY82.2  ZmWRKY83  ZmWRKY84.1  ZmWRKY84.2  ZmWRKY85  ZmWRKY86  ZmWRKY87  ZmWRKY88  ZmWRKY89  ZmWRKY90  ZmWRKY91  ZmWRKY92  ZmWRKY93  ZmWRKY94  ZmWRKY95  ZmWRKY96.1  ZmWRKY96.2  ZmWRKY97  ZmWRKY98  ZmWRKY99  ZmWRKY100  ZmWRKY101  ZmWRKY102  ZmWRKY103  ZmWRKY104  ZmWRKY105  ZmWRKY106*  ZmWRKY107  ZmWRKY108  ZmWRKY109.1  ZmWRKY109.2  ZmWRKY110  ZmWRKY111  ZmWRKY112  ZmWRKY113  ZmWRKY114  ZmWRKY115  ZmWRKY116.1  ZmWRKY116.2  ZmWRKY117  ZmWRKY118  ZmWRKY119  ZmWRKY120  ZmWRKY121.1  ZmWRKY121.2  ZmWRKY121.3  ZmWRKY122  ZmWRKY123  ZmWRKY124  ZmWRKY125 | Zm00001d028962_P001  Zm00001d029564_P001   Zm00001d030139_P001  Zm00001d030969_P001  Zm00001d032265_P001  Zm00001d033291_P007  Zm00001d033471_P001  Zm00001d033965_P001  Zm00001d034073_P002  Zm00001d034084_P001  Zm00001d034475_P001  Zm00001d034888_P001  Zm00001d002405_P001  Zm00001d002452_P001  Zm00001d002794_P001  Zm00001d002794_P002  Zm00001d003331_P001  Zm00001d004086_P001   Zm00001d005056_P001  Zm00001d005057_P001  Zm00001d005622_P001  Zm00001d005749_P001  Zm00001d006001_P001   Zm00001d006702_P001  Zm00001d007329_P001  Zm00001d039245_P003  Zm00001d039245_P002  Zm00001d039245_P001  Zm00001d039531_P001  Zm00001d039532_P001   Zm00001d039584_P001  Zm00001d040554_P001  Zm00001d041397_P001  Zm00001d041740_P001  Zm00001d041958_P001  GRMZM2G178671_P01  Zm00001d043025_P001  Zm00001d043060_P001  Zm00001d043062_P001   Zm00001d043063_P001  Zm00001d043066_P001  Zm00001d043474_P003  Zm00001d043474_P001  Zm00001d043569_P001  Zm00001d043663_P001  Zm00001d043950_P001  Zm00001d044010_P001  Zm00001d044162_P001  Zm00001d044171_P001  Zm00001d044315_P010  Zm00001d044682_P001  Zm00001d044680_P001  Zm00001d049173_P001  Zm00001d050023_P001  Zm00001d050195_P001  Zm00001d050247_P001  Zm00001d051328_P002  Zm00001d051550_P001  Zm00001d052357_P001   Zm00001d052358_P001  Zm00001d052355_P001  Zm00001d005056_P001  Zm00001d052847_P001  Zm00001d053369_P001  Zm00001d013307_P003  Zm00001d013630_P001  Zm00001d013709_P003  Zm00001d015515_P001  Zm00001d016052_P001  Zm00001d016457_P001  Zm00001d017712_P001  Zm00001d035323_P001  Zm00001d036726_P001   Zm00001d037054_P001  Zm00001d037607_P001  Zm00001d037607_P003  Zm00001d037854_P001   Zm00001d038023_P003   Zm00001d038023_P001  Zm00001d038451_P002  Zm00001d038761_P002  Zm00001d038761_P001   Zm00001d038843_P001  Zm00001d039032_P001  Zm00001d039044_P001  Zm00001d039044_P002  Zm00001d018656_P001  Zm00001d020136_P001  Zm00001d020137_P001  Zm00001d020492_P001  Zm00001d020492_P002  Zm00001d020495_P001  Zm00001d020881_P005  Zm00001d020881_P006  Zm00001d021947_P002  Zm00001d022437_P001  Zm00001d008190_P001  Zm00001d008578_P001  Zm00001d008793_P001  Zm00001d008794_P001  Zm00001d009595_P001  Zm00001d009619_P001  Zm00001d009698_P001  Zm00001d009939_P001  Zm00001d020137_P001  Zm00001d010399_P003  Zm00001d010399_P001  Zm00001d010616_P001  Zm00001d010617_P001  Zm00001d010805_P001  Zm00001d011133_P001  Zm00001d011237_P001  Zm00001d011403_P001  Zm00001d011413_P001  Zm00001d011496_P002   Zm00001d011527_P001  GRMZM2G045560_P01  Zm00001d012482_P001  Zm00001d012505_P001  Zm00001d012508_P001  Zm00001d012508_P003  Zm00001d012507_P001  Zm00001d012746_P001  Zm00001d012789_P001  Zm00001d045283_P001  Zm00001d045375_P001  Zm00001d046805_P001  Zm00001d047309_P002  Zm00001d047309_P001  Zm00001d047574_P006  Zm00001d023332_P001  Zm00001d023336_P001  Zm00001d023615_P001  Zm00001d024323_P003  Zm00001d024323_P002  Zm00001d024323_P001  Zm00001d024376_P001  Zm00001d025669_P003  Zm00001d026218_P001  Zm00001d026252_P001 |  | 53030837-53032126  76584638-76589606  108483337-108484748  169641684-169643906  219763429-219764430  257763592-257766730  263620528-263621882  279763671-279766568  283194695-283197425  283648741-283651017  294012381-294013358  304812196-304817687  12046655-12047909  13068292-13071803  21500615-21505542  21500684-21505542  40361661-40367040  81414319-81415921  156393219-156394669  156441979-156443513  180965628-180967221  186040975-186042528  195047188-195051225  214685549-214691851  228732533-228737268  474649-476986  474649-476986  474649-476986  7394664-7395768  7399394-7401753  8455756-8463632  49328805-49332591  116329475-116332429  135595517-135603519  145661117-145663100  180598307-180598998  186840855-186843842  187829245-187830523  187858083-187861043  187943172-187952706  188050986-188055909  201316396-201317932  201316331-201317960  204199572-204201279  206352165-206353102  214609031-214612175  216438639-216439909  220824499-220827409  221069092-221071008  225036822-225038634  234788638-234791964  234675094-234677643  19025176-19028095  60696494-60714980  72636377-72637297  76138362-76139324  154581238-154589610  162305826-162310127  188104225-188107989  188123494-188124602  188043994-188045262  156393219-156394669  202445149-202447772  227812359-227814181  8180183-8183770  15863794-15865837  18224234-18227041  95183890-95185688  141693514-141695426  162995367-162996627  204965594-204969901  21265695-21270357  99175591-99177057  110670329-110671228  127590620..127592172  127590620-127592125  139821260-139824197  141413048-141415248  141413610-141415248  157439807-157442874  163995859-163996996  163995859-163996782  165655159-165656827  168945257-168948458  169167038-169169532  169167433-165169532  2050308-2051829  95252065-95254096  95348911-95350784  115308636-115310466  115308709-115310466  118963352-118964584  135317347-135321114  135317350-135321287  166771846-166782537  177695470-177698120  774348-784221  13459466-13460938  20528476-20529755  20545295-20547560  72169414-72173597  73563517-73564793  76319744-76321343  90768544-90769854  95348911-95350784  110214059-110216271  110214059-110216431  121973116-121975989  122038132-122039596  128361517-128362582  140010389-140011159  144154120-144155223  149539154-149540733  149844919-149847262  152419720-152421768  153565171-153568905  168823637-168828313  175328253-175331243  175577410-175578506  170384146-170386361  170359322-170386361  175594122-175596874  179620170-179621660  181008342-181010034  17935946-17939379  20006174-20007083  106603130-106604806  124229921-124235305  124229921-124236498  136089588-136091036  3599571-3601619  3631087-3632656  12201891-12203553  65416247-65418035  65416688-65418035  65416943-65418035  67963922-67964857  125756468-125761380  141366825-141369972  142052895-142054459 | 645  1134  1323  1125  1002  1065  300  1194  996  1281  978  1143  897  1518  855  312  2112  996  906  1053  996  1056  1704  1833  663  861  1233  1626  822  1644  882  1746  1104  1485  1014  546  1668  861  1002  792  723  720  921  864  636  1146  1008  1059  1038  516  765  1086  690  1701  921  963  708  1401  687  918  1125  906  2190  1110  1188  423  1110  1044  1152  978  1455  1491  1035  756  588  579  1230  765  1470  1092  657  1005  666  1227  1887  1680  999  909  975  909  648  801  993  1752  1842  1017  1524  1026  690  1623  1833  774  1044  618  975  426  1497  687  954  690  591  897  1050  1080  984  1245  1032  1782  795  714  729  1059  834  684  1191  753  1056  1239  1494  636  975  861  1146  750  606  666  936  1992  1314  951 | 214  377  440  374  333  354  99  397  331  426  325  380  298  505  284  103  703  331  301  350  331  351  567  610  220  286  410  541  273  547  293  581  367  494  337  181  555  286  333  263  240  239  306  287  211  381  335  352  345  171  254  361  229  566  306  320  235  466  228  305  374  301  729  369  395  140  369  347  383  325  484  496  344  251  195  192  409  254  489  363  218  334  221  408  628  559  332  302  324  302  215  266  330  583  613  338  507  341  229  540  610  257  347  205  324  141  498  228  317  229  196  298  349  359  327  414  343  593  264  237  242  352  277  227  396  250  351  412  497  211  324  286  381  249  201  221  311  663  437  316 | 23275.7  40097  47187.4  39509.9  35117.1  38044.1  11218.7  42797.9  34512  45111.8  33848  40887.7  31360.8  53053.1  30667.8  12024.6  74817.3  35383.9  32596.4  37606.2  34781.8  37649.3  60340.8  65678  24267.5  30191.1  43583.8  56667  28090  56206.6  31182.8  60611.7  39340.8  52528.7  36825.1  19460.57  59201.5  30423.7  35834.9  29686.4  26821.8  26155.2  33185.5  29671.9  22647.3  40180.8  34863  38331.3  36448.6  18163.1  28313.8  38551.3  24730.2  61709.9  31913.2  34317.4  25642.8  49090.7  25240.9  33982.3  39912.9   32596.4  78734.7  39342.8  42310.3  15122.5  39393.5  37643.71  40762.7  34405  50805.4  52619  36579.1  27009.4  21165.5  21743.9  43353.4  26744.3  51502.7  38042.7  23442.5  35155  22963.4  42835.6  66901.9  59415.4  34764.7  32355.9  34355.2  32496.5  23275.1  28958.6  35233  62284.9  65877  34667.4  53194  36589.8  24138.9  55471.6  64641.6  27683.1  37267.4  22045.1  34355.2  15041.7  52522.7  25360.5  34473.6  24295.5  20552.6  30992.1  37362  39228.3  34595.8  43413.5  35977.97  62785.6  27603.7  26399.6  27416.6   37821.2  30023  25163.2  42365.9  27697.9  37959.6  43646.9  52589.6  23027.4  34391.6  32343.4  40293.6  27557.2  22412.3  24678  31888.7  70628.2  45881.9  32898.6 | 8.8414  7.0089  6.6522  10.6165  6.8913  10.3114  10.0582  10.5926  6.403  6.658  9.7671  7.1192  10.7007  5.0789  8.2009  9.5634  6.7853  10.3832  6.0719  6.4157  6.5472  8.9034  6.3932  6.8903  9.512  9.1218  7.7697  7.2802  6.2179  6.8915  9.8283  8.0288  10.6693  7.0345  5.5232  6.15  6.7381  6.8807  5.8695  5.1303  6.7245  4.9368  4.5780  5.2024  8.1378  7.2239  7.2149  7.2629  7.2372  5.8554  6.3278  6.0971  9.9793  6.6071  10.1958  6.3550  7.9228  6.2570  9.4185  6.9951  5.9479  6.0719  7.2974  5.0362  10.5304  6.0983  10.3755   8.3127  5.4729  9.1115  6.0964  7.8052  9.6831  8.9689   7.8268  9.5437  7.4141  7.595   7.9795  5.1305  9.2371  9.3773  7.1150  6.9909  7.3657  6.7207  6.8794  5.7894  6.7706  7.4763  9.1883  6.9457  6.6350  6.7588  6.5366  8.3431  9.1438  7.2555  7.6765  7.4069  6.6181  6.6455  8.0476  6.9578  6.7706  8.786  7.5613  7.4781  4.5514  8.4480  8.8811  9.4780  8.0091  6.7848  7.2266  6.5444  5.92  6.8121  6.0769  4.8512  5.9791  5.8651  4.6027  8.1522  6.0530  6.4963  9.9794  6.9705  7.1969  8.6911  6.3676  6.8667  5.8292  8.4822  8.583  8.6468  9.8892  7.0730  4.6916  10.7787 | 1  1  1  1  1  1  1  1  1  1  1  1  2  2  2  2  2  2  2  2  2  2  2  2  2  3  3  3  3  3  3  3  3  3  3  3  3  3  3  3  3  3  3  3  3  3  3  3  3  3  3  3  4  4  4  4  4  4  4  4  4  4  4  4  5  5  5  5  5  5  5  6  6  6  6  6  6  6  6  6  6  6  6  6  6  6  7  7  7  7  7  7  7  7  7  7  8  8  8  8  8  8  8  8  8  8  8  8  8  8  8  8  8  8  8  8  8  8  8  8  8  8  8  8  9  9  9  9  9  9  10  10  10  10  10  10  10  10  10  10 | III  I  IIe  IId  III  IId  IIc  IId  IIc  I  IId  IIe  IId  IIe  IIc  IIc  I  IId  III  III  III  IIa  I  I  IIc  IIb  IIb  IIb  IIc  IIb  IIc  IIb  IId  I  IIc  IIc  I  III  III  III  III  IIe  IIe  IIe  IIc  IIc  III  IIc  IIe  IIb  III  III  IIc  I  IId  III  IIc  IIe  III  III  III  III  I  IIe  IId  IIc  IId  IIa  IIe  IId  IIe  I  IIa  IIe  IIc  IIc  III  I  I  I  IIc  IIc  IIc  IIc  IIb  IIb  IIc  III  III  IIa  IIa  IIa  I  I  I  III  IIb  IIe  IIc  IIb  IIb  III  IIe  IIc  III  I  I  III  III  IIc  IIc  IIb  IIe  IIc  III  IIc  IIc  I  III  III  III  III  IIe  IIc  III  IIb  IIa  I  I  III  III  III  III  I  I  I  IId  I  IIe  IId |

**Note:** the information of maize WRKY gene family, including chromosomal location and ORF length, were retrieved from the B73 maize sequencing database (http://www.maize sequence.org/index.html).

*Indicates the gene which was not found in the latest B73_V4 genome but was annotated in B73_V3 genome.

**Table S2 Regular expressions of conserved motifs from ZmWRKY proteins**

| **Motif** | **E-value** | **Sites** | **Width** | **Multilevel consensus sequence** |
| --- | --- | --- | --- | --- |
| **1** | 1.1e-2992 | 136 | 29 | ILDDGYRWRKYGQKVIKGSPYPRSYYRCT |
| **2** | 8.6e-1855 | 108 | 29 | KGCPVRKQVZRSSDDPSVVITTYEGEHNH |
| **3** | 8.3e-384 | 14 | 39 | EDGYNWRKYGQKQVKGSENPRSYYKCTHPNCPVKKKVER |
| **4** | 2.1e-319 | 51 | 21 | AKGERPVREPRVAFQTRSEVD |
| **5** | 3.4e-248 | 21 | 29 | SADGQITEIVYKGAHNHPKPQKTRRKSSG |
| **6** | 6.9e-126 | 44 | 15 | KKRRKNRVKRVVRVP |
| **7** | 2.0e-124 | 16 | 29 | PSSYFTIPPGLSPATLLESPVLLSNSSSQ |
| **8** | 9.2e-122 | 25 | 21 | LEAELGRVNEENRRLEGMLTR |
| **9** | 3.7e-108 | 18 | 21 | YKDDQGCPATKQVQQKDSDDP |
| **10** | 1.8e-100 | 6 | 49 | GGSLPAGDNFLARAVLPCSSGVATISASAPFPTVTLDLTNPPPPCASSR |
| **11** | 1.3e-092 | 19 | 15 | PLFRVTYYNEHTCNS |
| **12** | 3.9e-076 | 15 | 29 | TESMAATJTSDPNFTAALAAAISSYLGEQ |
| **13** | 4.4e-075 | 13 | 21 | PWPTQRNALAGTTRNKPSSSS |
| **14** | 7.1e-070 | 7 | 30 | MEEVEEANRAAVESCHRVLALLSQPHDPAL |
| **15** | 6.0e-060 | 9 | 21 | PLPPAAVAMASTTSAAASMLL |
| **16** | 1.6e-052 | 14 | 21 | GEDDGDEDEPDSKRRKLELGA |
| **17** | 2.5e-068 | 17 | 21 | LAZVTAZAVAKFRKVINLLDR |
| **18** | 1.2e-054 | 3 | 49 | FSSFWPFPKFLHDFNATCSPTITFPQETELIRPKATRLASLPGDLPTQI |
| **19** | 6.4e-053 | 3  19 | 50  21 | DFGWFDQYPTWHRSALYAPLLPPEEWERELQGEDALFAGLGELPECAVVFDLCKQLASZILSCTDRSISAL |
| **20** | 8.4e-050 |  |  |  |

| **Table S3 Primers used in the paper.** | | |
| --- | --- | --- |
| **Constructions** |  | **Primer sequences** |
| **Primers for qRT-PCR analysis** | | |
| Actin-F |  | 5' CCTCACCGACCACCTAATG 3' |
| Actin-R |  | 5' CCATCAGGCATCTCGTAGC 3' |
| ZmWRKY11-F |  | 5' GCCGCCATTCTTTTCATTGAAA 3' |
| ZmWRKY11-R |  | 5' AAACAGACACGGAGAAACAGAA 3' |
| ZmWRKY13-F |  | 5' GCTAGGATTAGCTTCTCGTCTT 3' |
| ZmWRKY13-R |  | 5' CGATTTTCGTCTGAGCTTGTAG 3' |
| ZmWRKY17-F |  | 5' CTGCTCTGTCCTGTCAATTACT 3' |
| ZmWRKY17-R |  | 5' GAAAACGGAGCTGCTAAAACTG 3' |
| ZmWRKY30-F |  | 5' GTGGCTAAGAAGAACGCACCGATGC 3' |
| ZmWRKY30-R |  | 5' CTCTAATTTTATTATAGGTTTTTGC 3' |
| ZmWRKY51-F |  | 5' AGGGTTGAAAGCAAAGACTGACA 3' |
| ZmWRKY51-R |  | 5' TCCTAATCCACCCTCACAACAAT 3' |
| ZmWRKY63-F |  | 5' GTGAAGATGACTATCTCGGTGGTC 3' |
| ZmWRKY63-R |  | 5' GCCTAACTCTCCGGTGTAACCTAG 3' |
| ZmWRKY122-F |  | 5' ACCAACAAGAATGCCTTCCA 3' |
| ZmWRKY122-R |  | 5' CCGCATCCGATACACTCTTT 3' |
| ZmWRKY125-F |  | 5' GTCTACGGTGGGCGAATCAT 3' |
| ZmWRKY125-R |  | 5' TTTCCAAGCTTAGCGTGAAGGT 3' |
| ZmWRKY5-F |  | 5' TCAAGACCTAACCGGGTTGCCGGATC 3' |
| ZmWRKY5-R |  | 5' TCTCTAGCATTCGCGATTCCATTATCG 3' |
| ZmWRKY20-F |  | 5' TACGACGAGTATAATGTGACCG 3' |
| ZmWRKY20-R |  | 5' CATCTCCTACGGTATCACCATC 3' |
| ZmWRKY35-F |  | 5' GAAGGACATCAACGGACACAAG 3' |
| ZmWRKY35-R |  | 5' GTAGTAGGCGATCTCGTACG 3' |
| ZmWRKY36-F |  | 5' GATGTCATGGACTACGATGTGA 3' |
| ZmWRKY36-R |  | 5' CATGTACCGTACGTATGTTTCG 3' |
| ZmWRKY55-F |  | 5' GTGCATCGAGTACTTCCATTTC 3' |
| ZmWRKY55-R |  | 5' GCTCCAGATACAAACTCCACTT 3' |
| ZmWRKY80-F |  | 5' TGCTGGATGTAATGTAACCGAT 3' |
| ZmWRKY80-R |  | 5' TGAACTGACAGCACTATTACGA 3' |
| ZmWRKY86-F |  | 5' CTCCTAGCAGCCTCGGATTC 3' |
| ZmWRKY86-R |  | 5' GATCGAAGCCAAACAGAAACAG 3' |
| ZmWRKY92-F |  | 5' GGAGTTCGAGACCCTGTTC 3' |
| ZmWRKY92-R |  | 5' TATCTAATTGGCGGCATAGAGG 3' |
| ZmWRKY97-F |  | 5' GGCGAGATATCCATGGCTATG 3' |
| ZmWRKY97-R |  | 5' ATGACCTCAGGCTCAGAGAAG 3' |
| ZmWRKY98-F |  | 5' GACGGTTTCGACAGATACTACT 3' |
| ZmWRKY98-R  ZmWRKY108-F  ZmWRKY108-R |  | 5' TCGAACTCTTGACGAAGAATCT 3'  5’ GATCCGGTAATGGAGTTCCTG 3’  5’ GAAACGTAGCGATGCCATGTAG 3’ |
| ZmWRKY119-F |  | 5' GTTGTTGACACAGCACACTATT 3' |
| ZmWRKY119-R |  | 5' CAAAGCTTTCATCCTGGATTCC 3' |
| ZmWRKY123-F |  | 5' GACGAACTGGAATCTAAACGAA 3' |
| ZmWRKY123-R |  | 5' CTCATGTCAATGGCGTATGACT 3' |
|  | | |

**Table S4 Ka/Ks analysis and divergence time estimated for maize duplicated WRKY paralogs**

| **No.** | **Gene I** | **Location** | **Gene II** | **Location** | **Type of**  **duplication** | **Ks** | **Ka** | **Ka/Ks** | **Duplication Date**  **(Mya)** |
| --- | --- | --- | --- | --- | --- | --- | --- | --- | --- |
|  |  |  |  |  |  |  |  |  |  |
| 1 | ZmWRKY1 | 1 | ZmWRKY117 | 9 | Segmental | 0.8223 | 0.4935 | 0.6001 | 63.2527 |
| 2 | ZmWRKY2 | 1 | ZmWRKY116 | 9 | Segmental | 0.2172 | 0.1109 | 0.5105 | 16.7089 |
| 3 | ZmWRKY4 | 1 | ZmWRKY30 | 3 | Segmental | 0.4649 | 0.0822 | 0.1769 | 35.7628 |
| 4 | ZmWRKY5 | 1 | ZmWRKY20 | 2 | Segmental | 0.6145 | 0.4764 | 0.7752 | 47.2724 |
| 5 | ZmWRKY6 | 1 | ZmWRKY30 | 3 | Segmental | 1.4639 | 0.2773 | 0.1894 | 112.6056 |
| 6 | ZmWRKY6 | 1 | ZmWRKY63 | 5 | Segmental | 0.33 | 0.0616 | 0.1867 | 25.3848 |
| 7 | ZmWRKY7 | 1 | ZmWRKY62 | 5 | Segmental | 0.1565 | 0.1146 | 0.7321 | 12.0397 |
| 8 | ZmWRKY8 | 1 | ZmWRKY61 | 5 | Segmental | 0.1256 | 0.0496 | 0.3944 | 9.6653 |
| 9 | ZmWRKY14 | 2 | ZmWRKY67 | 5 | Segmental | 0.8645 | 0.3498 | 0.4046 | 66.5007 |
| 10 | ZmWRKY15 | 2 | ZmWRKY53 | 4 | Segmental | 0.4988 | 0.0512 | 0.1026 | 38.3723 |
| 11 | ZmWRKY18 | 2 | ZmWRKY56 | 4 | Segmental | 1.3204 | 0.7551 | 0.5719 | 101.5706 |
| 12 | ZmWRKY19 | 2 | ZmWRKY55 | 4 | Segmental | 1.221 | 0.719 | 0.5888 | 93.9242 |
| 13 | ZmWRKY20 | 2 | ZmWRKY80 | 7 | Segmental | 0.4754 | 0.3479 | 0.7318 | 36.5659 |
| 14 | ZmWRKY22 | 2 | ZmWRKY59 | 4 | Segmental | 1.5501 | 0.3996 | 0.2578 | 119.2372 |
| 15 | ZmWRKY22 | 2 | ZmWRKY84 | 7 | Segmental | 0.1728 | 0.0696 | 0.4026 | 13.296 |
| 16 | ZmWRKY23 | 2 | ZmWRKY85 | 7 | Segmental | 0.1783 | 0.045 | 0.2524 | 13.7181 |
| 17 | ZmWRKY26 | 3 | ZmWRKY89 | 8 | Segmental | 0.2302 | 0.1297 | 0.5635 | 17.7092 |
| 18 | ZmWRKY27 | 3 | ZmWRKY90 | 8 | Segmental | 0.201 | 0.0996 | 0.4956 | 15.4623 |
| 19 | ZmWRKY29 | 3 | ZmWRKY87 | 8 | Segmental | 0.4829 | 0.1851 | 0.3834 | 37.1489 |
| 20 | ZmWRKY30 | 3 | ZmWRKY68 | 6 | Segmental | 2.0012 | 0.9901 | 0.4947 | 153.9372 |
| 21 | ZmWRKY34 | 3 | ZmWRKY74 | 6 | Segmental | 0.4335 | 0.2716 | 0.6265 | 33.3474 |
| 22 | ZmWRKY34 | 3 | ZmWRKY107 | 8 | Segmental | 0.1399 | 0.0645 | 0.4611 | 10.7613 |
| 23 | ZmWRKY35 | 3 | ZmWRKY108 | 8 | Segmental | 0.1823 | 0.0854 | 0.4682 | 14.025 |
| 24 | ZmWRKY36 | 3 | ZmWRKY110 | 8 | Segmental | 1.3278 | 0.6205 | 0.4673 | 102.1376 |
| 25 | ZmWRKY36 | 3 | ZmWRKY97 | 8 | Segmental | 1.2436 | 0.6923 | 0.5567 | 95.6587 |
| 26 | ZmWRKY38 | 3 | ZmWRKY109 | 8 | Segmental | 0.2291 | 0.0815 | 0.3556 | 17.6266 |
| 27 | ZmWRKY39 | 3 | ZmWRKY111 | 8 | Segmental | 0.1973 | 0.0924 | 0.4684 | 15.1762 |
| 28 | ZmWRKY41 | 3 | ZmWRKY76 | 6 | Segmental | 0.8514 | 0.314 | 0.3688 | 65.4954 |
| 29 | ZmWRKY42 | 3 | ZmWRKY77 | 6 | Segmental | 0.5791 | 0.3061 | 0.5286 | 44.5462 |
| 30 | ZmWRKY42 | 3 | ZmWRKY105 | 8 | Segmental | 0.2069 | 0.1208 | 0.5838 | 15.9146 |
| 31 | ZmWRKY43 | 3 | ZmWRKY104 | 8 | Segmental | 0.1988 | 0.1248 | 0.6275 | 15.2959 |
| 32 | ZmWRKY44 | 3 | ZmWRKY103 | 8 | Segmental | 0.2057 | 0.0978 | 0.4756 | 15.8252 |
| 33 | ZmWRKY45 | 3 | ZmWRKY93 | 8 | Segmental | 0.7401 | 0.4144 | 0.56 | 56.9274 |
| 34 | ZmWRKY45 | 3 | ZmWRKY102 | 8 | Segmental | 0.1995 | 0.1168 | 0.5856 | 15.344 |
| 35 | ZmWRKY46 | 3 | ZmWRKY101 | 8 | Segmental | 0.3473 | 0.2972 | 0.8557 | 26.7157 |
| 36 | ZmWRKY54 | 4 | ZmWRKY67 | 5 | Segmental | 0.2753 | 0.1185 | 0.4303 | 21.1787 |
| 37 | ZmWRKY59 | 4 | ZmWRKY84 | 7 | Segmental | 1.8761 | 0.403 | 0.2148 | 144.3168 |
| 38 | ZmWRKY64 | 5 | ZmWRKY69 | 6 | Segmental | 0.6755 | 0.3668 | 0.5429 | 51.965 |
| 39 | ZmWRKY69 | 6 | ZmWRKY115 | 9 | Segmental | 0.2455 | 0.1577 | 0.6423 | 18.8817 |
| 40 | ZmWRKY74 | 6 | ZmWRKY107 | 8 | Segmental | 0.5035 | 0.2906 | 0.5771 | 38.7312 |
| 41 | ZmWRKY75 | 6 | ZmWRKY99 | 8 | Segmental | 0.3459 | 0.1227 | 0.3546 | 26.6089 |
| 42 | ZmWRKY75 | 6 | ZmWRKY112 | 8 | Segmental | 0.9557 | 0.4365 | 0.4567 | 73.5178 |
| 43 | ZmWRKY76 | 6 | ZmWRKY100 | 8 | Segmental | 0.2194 | 0.1186 | 0.5407 | 16.8751 |
| 44 | ZmWRKY77 | 6 | ZmWRKY105 | 8 | Segmental | 0.6035 | 0.3548 | 0.588 | 46.4224 |
| 45 | ZmWRKY78 | 6 | ZmWRKY91 | 8 | Segmental | 0.2463 | 0.138 | 0.5602 | 18.946 |
| 46 | ZmWRKY92 | 8 | ZmWRKY104 | 8 | Segmental | 0.6745 | 0.5087 | 0.7541 | 51.8873 |
| 47 | ZmWRKY93 | 8 | ZmWRKY102 | 8 | Segmental | 0.8022 | 0.4283 | 0.5339 | 61.7114 |
| 48 | ZmWRKY98 | 8 | ZmWRKY108 | 8 | Segmental | 0.9435 | 0.5941 | 0.6297 | 72.5752 |
| 49 | ZmWRKY99 | 8 | ZmWRKY112 | 8 | Segmental | 0.9403 | 0.4399 | 0.4679 | 72.332 |
| 50 | ZmWRKY122 | 10 | ZmWRKY13 | 2 | Segmental | 0.9174 | 0.33 | 0.3597 | 70.5684 |
| 51 | ZmWRKY123 | 10 | ZmWRKY16 | 2 | Segmental | 0.2024 | 0.0996 | 0.492 | 15.5709 |
| 52 | ZmWRKY124 | 10 | ZmWRKY14 | 2 | Segmental | 0.2903 | 0.0646 | 0.2225 | 22.3331 |

**Table S5 Duplicated WRKY orthologs between maize and *Arabidopsis***

| **Gene name** | **Chr.** | **Genome Location** | | **Gene name** | **Chr.** | **Genome Location** | |
| --- | --- | --- | --- | --- | --- | --- | --- |
| Zm00001d043025 | Zm3 | 186840855 | 186843842 | AT2G30250 | At2 | 12903208 | 12905338 |
| Zm00001d043063 | Zm3 | 187943172 | 187952706 | AT5G01900 | At5 | 350925 | 352095 |
| Zm00001d052357 | Zm4 | 188104225 | 188107989 | AT5G01900 | At5 | 350925 | 352095 |
| Zm00001d052847 | Zm4 | 202445149 | 202447772 | AT5G56270 | At5 | 22779614 | 22783426 |
| Zm00001d038451 | Zm6 | 157439807 | 157442874 | AT2G30250 | At2 | 12903208 | 12905338 |
| Zm00001d012482 | Zm8 | 175328253 | 175331243 | AT2G30250 | At2 | 12903208 | 12905338 |

**Table S6 Duplicated WRKY orthologs between maize and rice**

| **Gene name** | **Chr.** | **Genome Location** | | **Gene name** | **Chr.** | **Genome Location** | |
| --- | --- | --- | --- | --- | --- | --- | --- |
| Zm00001d028962 | Zm1 | 53030837 | 53032126 | LOC_Os03g20550 | Os3 | 11650824 | 11652144 |
| Zm00001d029564 | Zm1 | 76584638 | 76589606 | LOC_Os07g40570 | Os7 | 24311898 | 24315383 |
| Zm00001d030139 | Zm1 | 108483337 | 108484748 | LOC_Os10g42850 | Os10 | 23107503 | 23108646 |
| Zm00001d030969 | Zm1 | 169641684 | 169643906 | LOC_Os12g40570 | Os12 | 25100479 | 25104175 |
| Zm00001d032265 | Zm1 | 219763429 | 219764430 | LOC_Os08g29660 | Os8 | 18220041 | 18222408 |
| Zm00001d033291 | Zm1 | 257763592 | 257766730 | LOC_Os12g40570 | Os12 | 25100479 | 25104175 |
| Zm00001d033471 | Zm1 | 263620528 | 263621882 | LOC_Os03g45450 | Os3 | 25651039 | 25652125 |
| Zm00001d033965 | Zm1 | 279763671 | 279766568 | LOC_Os03g53050 | Os3 | 30422191 | 30425543 |
| Zm00001d034073 | Zm1 | 283194695 | 283197425 | LOC_Os03g55080 | Os3 | 31323190 | 31326926 |
| Zm00001d034475 | Zm1 | 294012381 | 294013358 | LOC_Os03g58420 | Os3 | 33286932 | 33288483 |
| Zm00001d034888 | Zm1 | 304812196 | 304817687 | LOC_Os03g63810 | Os3 | 36039164 | 36043822 |
| Zm00001d002405 | Zm2 | 12046655 | 12047909 | LOC_Os04g51560 | Os4 | 30545175 | 30546577 |
| Zm00001d002405 | Zm2 | 12046655 | 12047909 | LOC_Os08g13840 | Os8 | 8258575 | 8259595 |
| Zm00001d002452 | Zm2 | 13068292 | 13071803 | LOC_Os02g47060 | Os2 | 28726783 | 28730933 |
| Zm00001d002452 | Zm2 | 13068292 | 13071803 | LOC_Os04g50920 | Os4 | 30132491 | 30136547 |
| Zm00001d002794 | Zm2 | 21500684 | 21505542 | LOC_Os04g46060 | Os4 | 27284275 | 27290983 |
| Zm00001d003331 | Zm2 | 40361661 | 40367040 | LOC_Os04g39570 | Os4 | 23579869 | 23587188 |
| Zm00001d005056 | Zm2 | 156393219 | 156394669 | LOC_Os11g02520 | Os11 | 782358 | 783511 |
| Zm00001d005056 | Zm2 | 156393219 | 156394669 | LOC_Os12g02450 | Os12 | 824302 | 825793 |
| Zm00001d005057 | Zm2 | 156441979 | 156443513 | LOC_Os11g02470 | Os11 | 749998 | 751210 |
| Zm00001d005057 | Zm2 | 156441979 | 156443513 | LOC_Os12g02400 | Os12 | 789403 | 790687 |
| Zm00001d005749 | Zm2 | 186040975 | 186042528 | LOC_Os09g25060 | Os9 | 14975932 | 14977713 |
| Zm00001d006001 | Zm2 | 195047188 | 195051225 | LOC_Os09g30400 | Os9 | 18496949 | 18501264 |
| Zm00001d006702 | Zm2 | 214685549 | 214691851 | LOC_Os07g39480 | Os7 | 23654076 | 23659625 |
| Zm00001d039245 | Zm3 | 474649 | 476986 | LOC_Os01g14440 | Os1 | 8084372 | 8087044 |
| Zm00001d039531 | Zm3 | 7394664 | 7395768 | LOC_Os01g09100 | Os1 | 4572448 | 4573409 |
| Zm00001d039532 | Zm3 | 7399394 | 7401753 | LOC_Os01g09080 | Os1 | 4566304 | 4568617 |
| Zm00001d039584 | Zm3 | 8455756 | 8463632 | LOC_Os01g08710 | Os1 | 4340849 | 4356383 |
| Zm00001d040554 | Zm3 | 49328805 | 49332591 | LOC_Os01g18584 | Os1 | 10470540 | 10478800 |
| Zm00001d040554 | Zm3 | 49328805 | 49332591 | LOC_Os05g04640 | Os5 | 2179520 | 2184940 |
| Zm00001d041397 | Zm3 | 116329475 | 116332429 | LOC_Os12g40570 | Os12 | 25100479 | 25104175 |
| Zm00001d041740 | Zm3 | 135595517 | 135603519 | LOC_Os12g32250 | Os12 | 19473728 | 19478606 |
| Zm00001d041958 | Zm3 | 145661117 | 145663100 | LOC_Os01g74140 | Os1 | 42946753 | 42948750 |
| Zm00001d043025 | Zm3 | 186840855 | 186843842 | LOC_Os01g61080 | Os1 | 35347978 | 35350645 |
| Zm00001d043025 | Zm3 | 186840855 | 186843842 | LOC_Os05g39720 | Os5 | 23310474 | 23313449 |
| Zm00001d043060 | Zm3 | 187829245 | 187830523 | LOC_Os01g60640 | Os1 | 35062734 | 35064940 |
| Zm00001d043060 | Zm3 | 187829245 | 187830523 | LOC_Os05g40060 | Os5 | 23529423 | 23530499 |
| Zm00001d043062 | Zm3 | 187858083 | 187861043 | LOC_Os01g60540 | Os1 | 35008866 | 35011098 |
| Zm00001d043062 | Zm3 | 187858083 | 187861043 | LOC_Os05g40070 | Os5 | 23536113 | 23539013 |
| Zm00001d043063 | Zm3 | 187943172 | 187952706 | LOC_Os01g60520 | Os1 | 34996792 | 35003226 |
| Zm00001d043066 | Zm3 | 188050986 | 188055909 | LOC_Os01g60490 | Os1 | 34981468 | 34985447 |
| Zm00001d043474 | Zm3 | 201316396 | 201317932 | LOC_Os01g54600 | Os1 | 31409004 | 31410978 |
| Zm00001d043569 | Zm3 | 204199572 | 204201279 | LOC_Os01g53040 | Os1 | 30480134 | 30482161 |
| Zm00001d043663 | Zm3 | 206352165 | 206353102 | LOC_Os01g51690 | Os1 | 29720923 | 29723065 |
| Zm00001d043663 | Zm3 | 206352165 | 206353102 | LOC_Os05g46020 | Os5 | 26682472 | 26684208 |
| Zm00001d043950 | Zm3 | 214609031 | 214612175 | LOC_Os01g47560 | Os1 | 27196417 | 27200990 |
| Zm00001d043950 | Zm3 | 214609031 | 214612175 | LOC_Os05g49100 | Os5 | 28154693 | 28157989 |
| Zm00001d044010 | Zm3 | 216438639 | 216439909 | LOC_Os01g46800 | Os1 | 26687377 | 26688416 |
| Zm00001d044010 | Zm3 | 216438639 | 216439909 | LOC_Os05g49620 | Os5 | 28471802 | 28473061 |
| Zm00001d044162 | Zm3 | 220824499 | 220827409 | LOC_Os01g43650 | Os1 | 25009453 | 25012236 |
| Zm00001d044162 | Zm3 | 220824499 | 220827409 | LOC_Os05g50610 | Os5 | 29009136 | 29013100 |
| Zm00001d044171 | Zm3 | 221069092 | 221071008 | LOC_Os01g43550 | Os1 | 24945282 | 24947296 |
| Zm00001d044171 | Zm3 | 221069092 | 221071008 | LOC_Os05g50700 | Os5 | 29064582 | 29065815 |
| Zm00001d049173 | Zm4 | 19025176 | 19028095 | LOC_Os11g29870 | Os11 | 17352085 | 17355820 |
| Zm00001d050195 | Zm4 | 72636377 | 72637297 | LOC_Os04g51560 | Os4 | 30545175 | 30546577 |
| Zm00001d050195 | Zm4 | 72636377 | 72637297 | LOC_Os08g13840 | Os8 | 8258454 | 8259597 |
| Zm00001d050247 | Zm4 | 76138362 | 76139324 | LOC_Os08g29660 | Os8 | 18220041 | 18222408 |
| Zm00001d051328 | Zm4 | 154581238 | 154589610 | LOC_Os02g43560 | Os2 | 26280253 | 26283914 |
| Zm00001d051328 | Zm4 | 154581238 | 154589610 | LOC_Os04g46060 | Os4 | 27284275 | 27290983 |
| Zm00001d051550 | Zm4 | 162305826 | 162310127 | LOC_Os02g47060 | Os2 | 28726783 | 28730933 |
| Zm00001d052357 | Zm4 | 188104225 | 188107989 | LOC_Os11g02470 | Os11 | 749998 | 751210 |
| Zm00001d052357 | Zm4 | 188104225 | 188107989 | LOC_Os12g02400 | Os12 | 789403 | 790687 |
| Zm00001d052358 | Zm4 | 188123494 | 188124602 | LOC_Os11g02520 | Os11 | 782358 | 783511 |
| Zm00001d052358 | Zm4 | 188123494 | 188124602 | LOC_Os12g02440 | Os12 | 817003 | 823002 |
| Zm00001d005056 | Zm4 | 156393219 | 156394669 | LOC_Os12g02450 | Os12 | 824302 | 825793 |
| Zm00001d052847 | Zm4 | 202445149 | 202447772 | LOC_Os08g38990 | Os8 | 24645860 | 24649085 |
| Zm00001d052847 | Zm4 | 202445149 | 202447772 | LOC_Os09g30400 | Os9 | 18496949 | 18500439 |
| Zm00001d053369 | Zm4 | 227812359 | 227814181 | LOC_Os02g16540 | Os2 | 9446492 | 9448847 |
| Zm00001d013307 | Zm5 | 8180183 | 8183770 | LOC_Os03g53050 | Os3 | 30422191 | 30425543 |
| Zm00001d013630 | Zm5 | 15863794 | 15865837 | LOC_Os03g45450 | Os3 | 25651039 | 25652125 |
| Zm00001d013630 | Zm5 | 15863794 | 15865837 | LOC_Os02g08440 | Os2 | 4542762 | 4544983 |
| Zm00001d013630 | Zm5 | 15863794 | 15865837 | LOC_Os06g44010 | Os6 | 26509919 | 26511699 |
| Zm00001d016052 | Zm5 | 141693514 | 141695426 | LOC_Os02g16540 | Os2 | 9446492 | 9448847 |
| Zm00001d016457 | Zm5 | 162995367 | 162996627 | LOC_Os02g26430 | Os2 | 15521497 | 15522724 |
| Zm00001d017712 | Zm5 | 204965594 | 204969901 | LOC_Os10g42850 | Os10 | 23107503 | 23108646 |
| Zm00001d017712 | Zm5 | 204965594 | 204969901 | LOC_Os02g47060 | Os2 | 28726783 | 28730933 |
| Zm00001d017712 | Zm5 | 204965594 | 204969901 | LOC_Os04g50920 | Os4 | 30132491 | 30136547 |
| Zm00001d035323 | Zm6 | 21265695 | 21270357 | LOC_Os12g32250 | Os12 | 19473728 | 19478606 |
| Zm00001d036726 | Zm6 | 99175591 | 99177057 | LOC_Os02g08440 | Os2 | 4542762 | 4544983 |
| Zm00001d036726 | Zm6 | 99175591 | 99177057 | LOC_Os06g44010 | Os6 | 26509919 | 26511699 |
| Zm00001d037607 | Zm6 | 127590620 | 127592125 | LOC_Os05g09020 | Os5 | 4998210 | 4999629 |
| Zm00001d038023 | Zm6 | 141413610 | 141415248 | LOC_Os05g27730 | Os5 | 16150266 | 16152747 |
| Zm00001d038451 | Zm6 | 157439807 | 157442874 | LOC_Os01g61080 | Os1 | 35347978 | 35350645 |
| Zm00001d038451 | Zm6 | 157439807 | 157442874 | LOC_Os05g39720 | Os5 | 23310474 | 23313449 |
| Zm00001d038761 | Zm6 | 163995859 | 163996996 | LOC_Os05g45230 | Os5 | 26256951 | 26257809 |
| Zm00001d038843 | Zm6 | 165655159 | 165656827 | LOC_Os01g51690 | Os1 | 29720923 | 29723065 |
| Zm00001d038843 | Zm6 | 165655159 | 165656827 | LOC_Os05g46020 | Os5 | 26682472 | 26684208 |
| Zm00001d039032 | Zm6 | 168945257 | 168948458 | LOC_Os01g47560 | Os1 | 27196417 | 27200990 |
| Zm00001d039032 | Zm6 | 168945257 | 168948458 | LOC_Os05g49100 | Os5 | 28154693 | 28157989 |
| Zm00001d039044 | Zm6 | 169167038 | 169169532 | LOC_Os05g49210 | Os5 | 28238562 | 28241041 |
| Zm00001d018656 | Zm7 | 2050308 | 2051829 | LOC_Os07g02060 | Os7 | 630562 | 634316 |
| Zm00001d020136 | Zm7 | 95252065 | 95254096 | LOC_Os09g16510 | Os9 | 10128825 | 10131136 |
| Zm00001d020492 | Zm7 | 115308636 | 115310466 | LOC_Os09g25060 | Os9 | 14975932 | 14977713 |
| Zm00001d020881 | Zm7 | 135317350 | 135321287 | LOC_Os09g30400 | Os9 | 18496949 | 18500579 |
| Zm00001d021947 | Zm7 | 166771846 | 166782537 | LOC_Os07g39480 | Os7 | 23654076 | 23659625 |
| Zm00001d022437 | Zm7 | 177695470 | 177698120 | LOC_Os03g21710 | Os3 | 12394669 | 12396898 |
| Zm00001d022437 | Zm7 | 177695470 | 177698120 | LOC_Os07g48260 | Os7 | 28828793 | 28832398 |
| Zm00001d008190 | Zm8 | 774348 | 784221 | LOC_Os01g18584 | Os1 | 10470540 | 10478800 |
| Zm00001d009595 | Zm8 | 72169414 | 72173597 | LOC_Os05g49210 | Os5 | 28238562 | 28241041 |
| Zm00001d009619 | Zm8 | 73563517 | 73564793 | LOC_Os05g49620 | Os5 | 28471802 | 28473061 |
| Zm00001d009698 | Zm8 | 76319744 | 76321343 | LOC_Os01g43550 | Os1 | 24945286 | 24947296 |
| Zm00001d009698 | Zm8 | 76319744 | 76321343 | LOC_Os05g50700 | Os5 | 29064582 | 29065815 |
| Zm00001d009939 | Zm8 | 90768544 | 90769854 | LOC_Os01g09100 | Os1 | 4572448 | 4573409 |
| Zm00001d009939 | Zm8 | 90768544 | 90769854 | LOC_Os05g09020 | Os5 | 4998210 | 4999626 |
| Zm00001d010616 | Zm8 | 121973116 | 121975989 | LOC_Os05g40070 | Os5 | 23536113 | 23539013 |
| Zm00001d010617 | Zm8 | 122038132 | 122039596 | LOC_Os01g60640 | Os1 | 35062734 | 35064940 |
| Zm00001d010805 | Zm8 | 128361517 | 128362582 | LOC_Os01g53260 | Os1 | 30604295 | 30608077 |
| Zm00001d010805 | Zm8 | 128361517 | 128362582 | LOC_Os05g45230 | Os5 | 26256951 | 26257809 |
| Zm00001d011237 | Zm8 | 144154120 | 144155223 | LOC_Os01g40430 | Os1 | 22824809 | 22827116 |
| Zm00001d011403 | Zm8 | 149539154 | 149540733 | LOC_Os01g43550 | Os1 | 24945286 | 24947296 |
| Zm00001d011403 | Zm8 | 149539154 | 149540733 | LOC_Os05g50700 | Os5 | 29064582 | 29065815 |
| Zm00001d011413 | Zm8 | 149844919 | 149847262 | LOC_Os01g43650 | Os1 | 25009453 | 25012236 |
| Zm00001d011413 | Zm8 | 149844919 | 149847262 | LOC_Os05g50610 | Os5 | 29010970 | 29013172 |
| Zm00001d011496 | Zm8 | 152419720 | 152421768 | LOC_Os01g46800 | Os1 | 26687377 | 26688416 |
| Zm00001d011496 | Zm8 | 152419720 | 152421768 | LOC_Os05g49620 | Os5 | 28471802 | 28473061 |
| Zm00001d011527 | Zm8 | 153565171 | 153568905 | LOC_Os05g49100 | Os5 | 28154693 | 28157989 |
| Zm00001d012482 | Zm8 | 175328253 | 175331243 | LOC_Os01g61080 | Os1 | 35347978 | 35350645 |
| Zm00001d012482 | Zm8 | 175328253 | 175331243 | LOC_Os05g39720 | Os5 | 23310474 | 23313449 |
| Zm00001d012505 | Zm8 | 175577410 | 175578506 | LOC_Os01g60640 | Os1 | 35062734 | 35064940 |
| Zm00001d012508 | Zm8 | 170384146 | 170386361 | LOC_Os01g60490 | Os1 | 34981468 | 34985447 |
| Zm00001d012508 | Zm8 | 170384146 | 170386361 | LOC_Os05g40070 | Os5 | 23536113 | 23539013 |
| Zm00001d012507 | Zm8 | 175594122 | 175596874 | LOC_Os01g60540 | Os1 | 35008866 | 35011098 |
| Zm00001d012746 | Zm8 | 179620170 | 179621660 | LOC_Os01g54600 | Os1 | 31409004 | 31410978 |
| Zm00001d012789 | Zm8 | 181008342 | 181010034 | LOC_Os01g53260 | Os1 | 30604295 | 30608077 |
| Zm00001d045283 | Zm9 | 17935946 | 17939379 | LOC_Os06g06360 | Os6 | 2958991 | 2963006 |
| Zm00001d046805 | Zm9 | 106603130 | 106604806 | LOC_Os02g08440 | Os2 | 4542762 | 4544983 |
| Zm00001d046805 | Zm9 | 106603130 | 106604806 | LOC_Os06g44010 | Os6 | 26509919 | 26511699 |
| Zm00001d024376 | Zm10 | 67963922 | 67964857 | LOC_Os04g51560 | Os4 | 30545175 | 30546577 |
| Zm00001d024376 | Zm10 | 67963922 | 67964857 | LOC_Os08g13840 | Os8 | 8258575 | 8259595 |
| Zm00001d025669 | Zm10 | 125756468 | 125761380 | LOC_Os04g39570 | Os4 | 23579837 | 23583320 |
| Zm00001d026218 | Zm10 | 141366825 | 141369972 | LOC_Os02g47060 | Os2 | 28726783 | 28730933 |
| Zm00001d026218 | Zm10 | 141366825 | 141369972 | LOC_Os04g50920 | Os4 | 30132491 | 30136547 |

**Table S7 The original data of expression profiles of *ZmWRKY*1-125 genes in 10 tissues**

| **Gene name** | **Seed** | **Root** | **Seedling** | **Stem** | **Shoot Tip** | **Silks** | **Leaf** | **Tassel** | **Husk** | **Endosperm** |
| --- | --- | --- | --- | --- | --- | --- | --- | --- | --- | --- |
| *ZmWRKY1* | 5.66 | 7.28 | 6.47 | 6.11 | 6.02 | 6.94 | 5.59 | 5.81 | 6.99 | 5.32 |
| *ZmWRKY2* | 10.63 | 11.09 | 11.1 | 10.02 | 10.26 | 9.78 | 9.86 | 9.09 | 9.24 | 10.63 |
| *ZmWRKY3* | 5.63 | 5.82 | 6.49 | 7.7 | 7.4 | 6.77 | 5.69 | 6.41 | 6.13 | 6.15 |
| *ZmWRKY4* | 6.57 | 6.97 | 7.08 | 7.18 | 7.2 | 7.3 | 7.4 | 7.31 | 7.62 | 6.97 |
| *ZmWRKY5* | 5.89 | 7.7 | 6.61 | 6.26 | 6.93 | 7.29 | 5.65 | 7.3 | 5.99 | 6.12 |
| *ZmWRKY6* | 10.09 | 11.53 | 10.5 | 10.59 | 10.6 | 11.06 | 11.18 | 10.92 | 10.92 | 10.86 |
| *ZmWRKY7* | 8.11 | 7.95 | 8.19 | 7.5 | 7.51 | 7.88 | 7.74 | 7.18 | 7.46 | 8.02 |
| *ZmWRKY8* | 9.24 | 11.2 | 9.69 | 10.98 | 11.01 | 8.85 | 10.24 | 10.35 | 10.84 | 10.76 |
| *ZmWRKY9* | 6.06 | 6.92 | 5.26 | 5.81 | 4.94 | 6.23 | 4.91 | 5.53 | 5.89 | 8.06 |
| *ZmWRKY10* | 5.52 | 5.49 | 5.83 | 5.4 | 5.69 | 5.98 | 5.72 | 5.78 | 5.4 | 5.9 |
| *ZmWRKY11* | 4.82 | 6.84 | 5.33 | 5.46 | 5.4 | 5.54 | 4.89 | 5.22 | 4.96 | 4.94 |
| *ZmWRKY12* | 6.17 | 5.05 | 8.39 | 10.03 | 10.17 | 7.11 | 10.51 | 7.55 | 10.02 | 6.24 |
| *ZmWRKY13* | 8.33 | 10.47 | 9.09 | 9.08 | 8.94 | 9.06 | 8.89 | 9.18 | 10.01 | 9.11 |
| *ZmWRKY14* | 9.24 | 11.78 | 6.92 | 7.7 | 6.99 | 7.57 | 6.47 | 8.34 | 7.28 | 6.81 |
| *ZmWRKY15.1* | 4.84 | 4.77 | 5 | 8.09 | 6.65 | 4.86 | 5.21 | 6.71 | 5.29 | 5.08 |
| *ZmWRKY15.2* | 5.11 | 4.97 | 5.34 | 8.21 | 6.53 | 5.38 | 5.37 | 6.93 | 5.5 | 5.33 |
| *ZmWRKY16* | 5.25 | 5.23 | 5.49 | 6.14 | 7.41 | 7.26 | 7.74 | 7.53 | 6.21 | 5.34 |
| *ZmWRKY17* | 8.87 | 8.31 | 7.64 | 7.37 | 6.96 | 7.65 | 8.15 | 7.87 | 8.24 | 8.74 |
| *ZmWRKY18* | 5.26 | 5.68 | 5.14 | 5.28 | 4.99 | 4.91 | 5.51 | 4.64 | 5.22 | 5.86 |
| *ZmWRKY19* | 5.51 | 8 | 5.48 | 5.92 | 5.56 | 5.92 | 5.19 | 5.53 | 5.46 | 5.59 |
| *ZmWRKY20* | 5.14 | 6.07 | 5.31 | 5.69 | 5.5 | 5.52 | 5.44 | 6.17 | 5.69 | 4.87 |
| *ZmWRKY21* | 5.35 | 5.7 | 5.22 | 5.48 | 5.48 | 6.01 | 5.32 | 5.61 | 5.45 | 5.75 |
| *ZmWRKY22* | 12.75 | 13.01 | 12.12 | 12.32 | 12.06 | 12.17 | 9.68 | 11.5 | 12.36 | 9.52 |
| *ZmWRKY23* | 13.16 | 13.31 | 12.69 | 13.26 | 13.18 | 13.32 | 13.39 | 13.82 | 13.3 | 13.35 |
| *ZmWRKY24* | 5.86 | 5.35 | 5.64 | 5.82 | 5.52 | 5.67 | 5.46 | 5.26 | 6.07 | 5.3 |
| *ZmWRKY26* | 5.95 | 5.86 | 5.83 | 6.1 | 6.36 | 6.69 | 5.63 | 5.84 | 5.95 | 6.35 |
| *ZmWRKY27* | 5.32 | 10.19 | 6.05 | 5.6 | 5.31 | 9.85 | 5.73 | 6.1 | 5.7 | 5.51 |
| *ZmWRKY28* | 8.39 | 8.38 | 8.7 | 11.92 | 12.28 | 7.46 | 9.35 | 10.38 | 9.06 | 6.15 |
| *ZmWRKY29* | 5.26 | 6.13 | 5.58 | 5.4 | 5.33 | 5.82 | 5.89 | 5.41 | 5.85 | 5.88 |
| *ZmWRKY30* | 10.77 | 10.69 | 10.85 | 9.71 | 10.28 | 11.55 | 11.07 | 10.68 | 12.66 | 10.66 |
| *ZmWRKY31* | 12.12 | 12.73 | 11.75 | 11.47 | 11.02 | 12.45 | 11.21 | 11.69 | 12.17 | 10.72 |
| *ZmWRKY33* | 6.06 | 6.17 | 6.64 | 6.18 | 6.3 | 6.8 | 6.58 | 5.85 | 6.16 | 6.34 |
| *ZmWRKY34* | 6.04 | 7.23 | 6.42 | 5.97 | 6.03 | 6.45 | 6.14 | 5.97 | 6.1 | 6.26 |
| *ZmWRKY35* | 5.27 | 6.77 | 5.69 | 5.85 | 5.65 | 6.45 | 6.51 | 5.64 | 6.25 | 6.06 |
| *ZmWRKY36* | 5.1 | 8.22 | 5.82 | 5.5 | 5.58 | 5.99 | 5.18 | 5.98 | 5.19 | 4.99 |
| *ZmWRKY37* | 5.83 | 6.88 | 5.87 | 5.71 | 5.63 | 5.68 | 5.05 | 5.35 | 5 | 7.27 |
| *ZmWRKY38* | 5.9 | 5.5 | 5.44 | 5.46 | 5.13 | 5.7 | 5.19 | 5.64 | 5.14 | 6 |
| *ZmWRKY39.1* | 7.34 | 10.17 | 7.5 | 7.25 | 7.03 | 8.49 | 7.67 | 6.31 | 8.89 | 7.27 |
| *ZmWRKY39.2* | 7.34 | 10.17 | 7.5 | 7.25 | 7.03 | 8.49 | 7.67 | 6.31 | 8.89 | 7.27 |
| *ZmWRKY40* | 7.53 | 11.44 | 8.37 | 7.36 | 7.04 | 11.39 | 6.71 | 6.33 | 11.41 | 7.2 |
| *ZmWRKY41* | 7.39 | 7.38 | 7.31 | 6.75 | 6.85 | 7.75 | 6.84 | 7.42 | 6.92 | 7.16 |
| *ZmWRKY42* | 9.19 | 8.13 | 8.32 | 7.57 | 7.56 | 8.57 | 7.4 | 7.81 | 9.32 | 7.6 |
| *ZmWRKY43* | 6.6 | 6.56 | 6.76 | 6.54 | 6.76 | 8 | 6.57 | 6.8 | 6.72 | 6.69 |
| *ZmWRKY44* | 5.72 | 5.48 | 6.07 | 5.91 | 5.69 | 6.21 | 6.64 | 5.76 | 6.14 | 5.55 |
| *ZmWRKY45* | 5.34 | 6.25 | 5.6 | 5.37 | 5.39 | 6.23 | 5.73 | 5.5 | 5.79 | 5.56 |
| *ZmWRKY46* | 5.11 | 5.5 | 5.47 | 5.48 | 5.67 | 6.08 | 5.6 | 5.67 | 5.83 | 5.66 |
| *ZmWRKY47* | 6.81 | 6.48 | 6.21 | 6.02 | 6.24 | 7.35 | 6.18 | 6.64 | 6.4 | 6.87 |
| *ZmWRKY48* | 5.89 | 5.6 | 5.63 | 5.42 | 5.57 | 5.75 | 6.37 | 5.96 | 7.01 | 5.86 |
| *ZmWRKY49* | 6.43 | 6.47 | 6.49 | 5.69 | 6.11 | 6.49 | 5.7 | 5.7 | 5.92 | 6.05 |
| *ZmWRKY50* | 11.9 | 11.99 | 11.5 | 11.86 | 12.12 | 12.79 | 12.34 | 12.55 | 12.3 | 11.96 |
| *ZmWRKY51* | 6.63 | 9.14 | 7.34 | 7.29 | 7.34 | 7.89 | 7.73 | 7.41 | 7.53 | 7.06 |
| *ZmWRKY52* | 5.24 | 8.9 | 5.89 | 5.58 | 6.01 | 5.98 | 5.43 | 6.12 | 5.59 | 5.36 |
| *ZmWRKY53* | 5.42 | 4.8 | 6.39 | 8.29 | 9.34 | 5.9 | 6.88 | 8.24 | 7.45 | 5.65 |
| *ZmWRKY54* | 7.33 | 8.62 | 6.7 | 7.68 | 7.14 | 6.11 | 7.31 | 6.38 | 8.53 | 6.15 |
| *ZmWRKY55* | 10.35 | 10.99 | 9.36 | 9.09 | 8.77 | 12.29 | 9.34 | 8.85 | 10.27 | 8.18 |
| *ZmWRKY56* | 6.64 | 4.95 | 5.03 | 5.08 | 4.98 | 5.36 | 5.3 | 5.49 | 5.37 | 6.34 |
| *ZmWRKY57* | 5.54 | 6.56 | 5.97 | 5.69 | 6.13 | 8.42 | 5.48 | 7.38 | 5.77 | 5.97 |
| *ZmWRKY58* | 5.14 | 8.28 | 5.83 | 5.59 | 5.57 | 5.55 | 5.47 | 5.15 | 5.33 | 5.35 |
| *ZmWRKY59* | 10.13 | 5.13 | 7.07 | 4.99 | 5.63 | 4.85 | 5.36 | 5.49 | 5.85 | 5.96 |
| *ZmWRKY60* | 6.98 | 9.14 | 7.78 | 8.36 | 8.51 | 8.44 | 8.22 | 7.8 | 8.75 | 6.59 |
| *ZmWRKY61* | 11.21 | 11.7 | 10.87 | 12.09 | 11.99 | 9.7 | 11.16 | 11.32 | 11.57 | 12.37 |
| *ZmWRKY62* | 7.53 | 6.41 | 7.04 | 6.37 | 6.65 | 7.17 | 6.83 | 6.5 | 6.58 | 6.91 |
| *ZmWRKY63* | 10.13 | 11.96 | 10.38 | 10.88 | 10.56 | 11.65 | 10.26 | 10.67 | 11.11 | 10.45 |
| *ZmWRKY64* | 9.17 | 10.4 | 6.99 | 5.92 | 6.09 | 7.02 | 5.61 | 6.46 | 5.79 | 5.66 |
| *ZmWRKY65* | 7.4 | 9.18 | 8.48 | 8.31 | 8.15 | 8.58 | 8.81 | 8.4 | 8.63 | 8.15 |
| *ZmWRKY66* | 6.2 | 7.33 | 6.54 | 6.37 | 6.05 | 6.65 | 6.75 | 6.3 | 6.62 | 6.45 |
| *ZmWRKY67* | 5.96 | 7.33 | 5.66 | 6.24 | 5.96 | 5.4 | 5.47 | 5.81 | 5.94 | 5.32 |
| *ZmWRKY68* | 12.67 | 13.25 | 12.19 | 11.77 | 11.41 | 13.02 | 11.74 | 12.03 | 12.97 | 11.63 |
| *ZmWRKY69* | 6.88 | 8.78 | 7.05 | 6.48 | 6.34 | 6.71 | 6.73 | 6.23 | 6.29 | 6.58 |
| *ZmWRKY70* | 5.95 | 6.68 | 6.35 | 6.29 | 6.27 | 7.18 | 6.11 | 5.98 | 5.91 | 6.36 |
| *ZmWRKY71.1* | 5.64 | 9.47 | 7.04 | 7.44 | 5.69 | 4.99 | 4.73 | 5.76 | 7.73 | 4.97 |
| *ZmWRKY71.2* | 5.64 | 9.47 | 7.04 | 7.44 | 5.69 | 4.99 | 4.73 | 5.76 | 7.73 | 4.97 |
| *ZmWRKY72* | 7.15 | 6.6 | 7.39 | 6.55 | 6.75 | 7.46 | 6.56 | 7.12 | 6.94 | 7.06 |
| *ZmWRKY73.1* | 6.37 | 8.63 | 6.46 | 5.99 | 5.81 | 7.04 | 6.43 | 6.25 | 6.73 | 6.28 |
| *ZmWRKY73.2* | 6.37 | 8.63 | 6.46 | 5.99 | 5.81 | 7.04 | 6.43 | 6.25 | 6.73 | 6.28 |
| *ZmWRKY74* | 6.1 | 7.09 | 6.65 | 6.45 | 6.63 | 8.27 | 6.29 | 6.87 | 6.29 | 6.07 |
| *ZmWRKY75.1* | 6.34 | 6.83 | 7.71 | 6.17 | 6.53 | 7.21 | 5.79 | 7.28 | 7.05 | 6.28 |
| *ZmWRKY75.2* | 6.34 | 6.83 | 7.71 | 6.17 | 6.53 | 7.21 | 5.79 | 7.28 | 7.05 | 6.28 |
| *ZmWRKY76* | 8.11 | 7.45 | 7.59 | 6.39 | 6.18 | 6.04 | 5.42 | 6.92 | 7.07 | 5.46 |
| *ZmWRKY77* | 7.31 | 7.68 | 7.15 | 6.69 | 5.29 | 6.82 | 5.45 | 5.55 | 8.08 | 6.01 |
| *ZmWRKY78.1* | 6.44 | 6.79 | 6.85 | 7.02 | 7.4 | 7.5 | 6.65 | 7.12 | 6.38 | 6.47 |
| *ZmWRKY78.2* | 6.25 | 6.63 | 6.53 | 6.59 | 6.91 | 6.97 | 6.26 | 6.49 | 5.88 | 6.25 |
| *ZmWRKY79* | 5.52 | 6.06 | 5.98 | 6.05 | 5.95 | 6.69 | 6.09 | 5.82 | 6.38 | 5.86 |
| *ZmWRKY80* | 8.78 | 9.36 | 7.49 | 9.3 | 7.67 | 6.68 | 7.65 | 9.5 | 9.26 | 6.31 |
| *ZmWRKY81* | 7.99 | 11.59 | 8.34 | 9.51 | 8.63 | 8.55 | 9.22 | 10.6 | 8.33 | 7.91 |
| *ZmWRKY82.1* | 6.07 | 6.34 | 6.09 | 5.95 | 6.08 | 5.94 | 6.27 | 6.59 | 5.83 | 7.78 |
| *ZmWRKY82.2* | 5.27 | 5.88 | 5.43 | 5.34 | 5.61 | 5.3 | 5.61 | 5.55 | 5.2 | 6.31 |
| *ZmWRKY83* | 6.17 | 6.52 | 6.47 | 6.04 | 6.35 | 6.8 | 5.93 | 6.48 | 6.36 | 6.35 |
| *ZmWRKY84.1* | 11.21 | 12.12 | 11.1 | 10.79 | 11.06 | 10.92 | 6.8 | 10.25 | 10.97 | 8.19 |
| *ZmWRKY84.2* | 11.21 | 12.12 | 11.1 | 10.79 | 11.06 | 10.92 | 6.8 | 10.25 | 10.97 | 8.19 |
| *ZmWRKY85* | 12.89 | 13.26 | 12.53 | 13.27 | 13.09 | 12.78 | 12.93 | 13.76 | 12.79 | 13.33 |
| *ZmWRKY86* | 5.91 | 7.58 | 5.97 | 6.23 | 6.36 | 6.1 | 5.54 | 5.92 | 5.72 | 5.74 |
| *ZmWRKY87* | 7.03 | 6.3 | 7.02 | 7.1 | 6.71 | 7.68 | 7.08 | 7.05 | 7.91 | 7.03 |
| *ZmWRKY88* | 5.61 | 6.63 | 5.78 | 5.67 | 6.13 | 7.1 | 6.19 | 6.33 | 6.28 | 5.71 |
| *ZmWRKY89* | 4.85 | 5.43 | 5.13 | 5.25 | 5.25 | 5.33 | 5.09 | 5.26 | 5.44 | 5.05 |
| *ZmWRKY90* | 5.47 | 9.52 | 6.11 | 5.89 | 5.59 | 7.42 | 5.71 | 5.91 | 5.95 | 5.86 |
| *ZmWRKY91* | 6 | 6.52 | 6.17 | 5.88 | 6.14 | 6.84 | 6.06 | 6.42 | 6.05 | 6.27 |
| *ZmWRKY92* | 5.27 | 5.6 | 5.95 | 5.96 | 5.89 | 5.5 | 6.09 | 5.27 | 5.71 | 5.69 |
| *ZmWRKY93* | 5.69 | 6.38 | 6.35 | 6.51 | 5.88 | 6.42 | 6.26 | 6.46 | 6.19 | 5.92 |
| *ZmWRKY94* | 6.34 | 9.3 | 7.44 | 6.64 | 6.37 | 6.99 | 6.32 | 6.59 | 6.49 | 6.12 |
| *ZmWRKY95* | 7.99 | 11.59 | 8.34 | 9.51 | 8.63 | 8.55 | 9.22 | 10.6 | 8.33 | 7.91 |
| *ZmWRKY96.1* | 8.25 | 10.76 | 7.44 | 6.24 | 5.76 | 8.29 | 6.15 | 7 | 6.02 | 6.2 |
| *ZmWRKY96.2* | 8.25 | 10.76 | 7.44 | 6.24 | 5.76 | 8.29 | 6.15 | 7 | 6.02 | 6.2 |
| *ZmWRKY97* | 5.03 | 5.82 | 5.25 | 5.2 | 5.32 | 5.68 | 5.49 | 5.6 | 5.11 | 5 |
| *ZmWRKY98* | 4.93 | 8.12 | 5.1 | 5.29 | 4.96 | 4.87 | 5 | 5.42 | 4.89 | 5.21 |
| *ZmWRKY99* | 6.53 | 7.33 | 8.13 | 6.5 | 6.8 | 8.26 | 6.25 | 7.39 | 7.41 | 6.74 |
| *ZmWRKY100* | 9.49 | 12.05 | 10.59 | 10.56 | 10.9 | 11.51 | 11.32 | 10.62 | 12.33 | 12.04 |
| *ZmWRKY101* | 4.88 | 5.74 | 5.34 | 5.16 | 5.13 | 6 | 5.24 | 5.3 | 5.3 | 5.06 |
| *ZmWRKY102* | 6.09 | 8.95 | 6.39 | 6.47 | 6.18 | 6.68 | 6.31 | 6.19 | 6.84 | 6.38 |
| *ZmWRKY103* | 5.59 | 5.48 | 6.29 | 6.04 | 5.94 | 5.96 | 5.86 | 5.92 | 6.75 | 5.63 |
| *ZmWRKY104* | 5.49 | 5.51 | 5.91 | 5.58 | 5.58 | 6.09 | 5.72 | 6.01 | 6.1 | 5.56 |
| *ZmWRKY105* | 9.4 | 10.44 | 9.49 | 8.86 | 8.35 | 9.73 | 8.57 | 8.07 | 10.64 | 8.78 |
| *ZmWRKY107* | 6.04 | 6.66 | 6.41 | 5.99 | 6.31 | 6.43 | 6 | 6.24 | 6.36 | 6.04 |
| *ZmWRKY108* | 5.34 | 7.8 | 5.68 | 6.2 | 5.83 | 6.06 | 6.56 | 5.94 | 6.61 | 6.02 |
| *ZmWRKY109.1* | 5.41 | 5.02 | 4.9 | 4.96 | 4.96 | 4.99 | 4.71 | 5 | 5.18 | 5.53 |
| *ZmWRKY109.2* | 5.43 | 5 | 4.94 | 4.91 | 4.79 | 5.01 | 4.8 | 4.89 | 4.89 | 5.4 |
| *ZmWRKY110* | 5.22 | 4.96 | 5.15 | 5.1 | 5.12 | 5.4 | 5.28 | 5.31 | 5.14 | 5.4 |
| *ZmWRKY111* | 6.5 | 11.03 | 8.35 | 6.99 | 6.68 | 7.48 | 6.84 | 6.36 | 8.98 | 7.36 |
| *ZmWRKY112* | 6.11 | 8.76 | 8.48 | 5.76 | 5.55 | 6.26 | 5.75 | 5.5 | 6.79 | 6 |
| *ZmWRKY113* | 4.99 | 5.29 | 5.62 | 5.35 | 5.24 | 5.81 | 5.79 | 5.4 | 5.6 | 5.27 |
| *ZmWRKY114* | 6.15 | 6.74 | 6.16 | 5.54 | 5.58 | 5.85 | 5.54 | 5.91 | 5.65 | 6.42 |
| *ZmWRKY115* | 5.47 | 7.72 | 5.46 | 5.35 | 5.36 | 5.75 | 5.58 | 5.2 | 5.31 | 5.41 |
| *ZmWRKY116.1* | 10.79 | 11.39 | 11.68 | 9.88 | 10.67 | 11.51 | 11.07 | 10.2 | 10.94 | 10.87 |
| *ZmWRKY116.2* | 10.79 | 11.39 | 11.68 | 9.88 | 10.67 | 11.51 | 11.07 | 10.2 | 10.94 | 10.87 |
| *ZmWRKY117* | 8.06 | 7.46 | 7.13 | 6.37 | 6.83 | 8.67 | 7.08 | 6.79 | 8.77 | 6.92 |
| *ZmWRKY118* | 6.54 | 6.56 | 6.72 | 6.92 | 6.8 | 6.57 | 10.35 | 8.69 | 7.17 | 6.62 |
| *ZmWRKY119* | 7.14 | 6.7 | 5.83 | 5.56 | 7.67 | 8.7 | 7.11 | 7.06 | 7.72 | 7.82 |
| *ZmWRKY120* | 7.56 | 10.34 | 8.2 | 10.75 | 9.32 | 9.51 | 11.62 | 11.27 | 10.15 | 7.3 |
| *ZmWRKY121.1* | 7.7 | 7.47 | 9.72 | 6.67 | 7.12 | 10.36 | 6.82 | 7.67 | 6.42 | 7.18 |
| *ZmWRKY121.2* | 7.65 | 6.98 | 10.09 | 6.6 | 6.82 | 10.65 | 6.92 | 7.77 | 6.68 | 7.15 |
| *ZmWRKY121.3* | 7.65 | 6.98 | 10.09 | 6.6 | 6.82 | 10.65 | 6.92 | 7.77 | 6.68 | 7.15 |
| *ZmWRKY122* | 6.53 | 8.81 | 7.84 | 7.44 | 7.49 | 7.75 | 8.18 | 7.12 | 7.53 | 7.24 |
| *ZmWRKY123* | 5.19 | 5.34 | 5.71 | 6.38 | 6.84 | 6.34 | 7.12 | 7.19 | 6.5 | 5.66 |
| *ZmWRKY124* | 6.42 | 8.97 | 5.43 | 5.88 | 5.64 | 6 | 5.01 | 6.19 | 6 | 5.26 |
| *ZmWRKY125* | 7.38 | 10.32 | 8.1 | 8.53 | 7.94 | 9.13 | 8.83 | 8.92 | 9.56 | 8.99 |

**Table S8** **Subcellular localization of WRKY proteins in maize**

| **Serial**  **NO.** | **Gene Name** | **Subcellular localization**  **predictor** | **Serial**  **NO.** | **Gene Name** | **Subcellular localization**  **predictor** |
| --- | --- | --- | --- | --- | --- |
| **1**  **2**  **3**  **4**  **5**  **6**  **7**  **8**  **9**  **10**  **11**  **12**  **13**  **14**  **15**  **16**  **17**  **18**  **19**  **20**  **21**  **22**  **23**  **24**  **25**  **26**  **27**  **28**  **29**  **30**  **31**  **32**  **33**  **34**  **35**  **36**  **37**  **38**  **39**  **40**  **41**  **42**  **43**  **44**  **45**  **46**  **47**  **48**  **49**  **50**  **51**  **52**  **53**  **54**  **55**  **56**  **57**  **58**  **59**  **60**  **61**  **62**  **63**  **64**  **65**  **66**  **67**  **68**  **69**  **70** | ZmWRKY1  ZmWRKY2  ZmWRKY3  ZmWRKY4  ZmWRKY5  ZmWRKY6  ZmWRKY7  ZmWRKY8  ZmWRKY9  ZmWRKY10  ZmWRKY11  ZmWRKY12  ZmWRKY13  ZmWRKY14  ZmWRKY15.1  ZmWRKY15.2  ZmWRKY16  ZmWRKY17  ZmWRKY18  ZmWRKY19  ZmWRKY20  ZmWRKY21  ZmWRKY22  ZmWRKY23  ZmWRKY24  ZmWRKY25.1  ZmWRKY25.2  ZmWRKY25.3  ZmWRKY26  ZmWRKY27  ZmWRKY28  ZmWRKY29  ZmWRKY30  ZmWRKY31  ZmWRKY32  ZmWRKY33*  ZmWRKY34  ZmWRKY35  ZmWRKY36  ZmWRKY37  ZmWRKY38  ZmWRKY39.1  ZmWRKY39.2  ZmWRKY40  ZmWRKY41  ZmWRKY42  ZmWRKY43  ZmWRKY44  ZmWRKY45  ZmWRKY46  ZmWRKY47  ZmWRKY48  ZmWRKY49  ZmWRKY50  ZmWRKY51  ZmWRKY52  ZmWRKY53  ZmWRKY54  ZmWRKY55  ZmWRKY56  ZmWRKY57  ZmWRKY58  ZmWRKY59  ZmWRKY60  ZmWRKY61  ZmWRKY62  ZmWRKY63  ZmWRKY64  ZmWRKY65  ZmWRKY66 | Nucleus  Nucleus  Nucleus  Nucleus  Nucleus  Nucleus  Mitochondria  Nucleus  Nucleus  Nucleus  Nucleus  Nucleus  Nucleus  Nucleus  Chloroplast  Nucleus  Nucleus  Nucleus  Nucleus  Nucleus  Nucleus  Chloroplast  Nucleus  Nucleus  Nucleus  Chloroplast  Nucleus  Extracellular  Nucleus  Nucleus  Nucleus  Nucleus  Nucleus  Nucleus  Nucleus  Nucleus  Nucleus  Nucleus  Nucleus  Nucleus  Nucleus  Chloroplast  Nucleus  Cytoplasmic  Chloroplast  Nucleus  Nucleus  Nucleus  Nucleus  Chloroplast  Cytoplasmic  Nucleus  Nucleus  Endoplasmic reticulum  Nucleus  Nucleus  Nucleus  Nucleus  Nucleus  Nucleus  Nucleus  Nucleus  Nucleus  Nucleus  Nucleus  Mitochondria  Nucleus  Chloroplast  Nucleus  Nucleus | **71**  **72**  **73**  **74**  **75**  **76**  **77**  **78**  **79**  **80**  **81**  **82**  **83**  **84**  **85**  **86**  **87**  **88**  **89**  **90**  **91**  **92**  **93**  **94**  **95**  **96**  **97**  **98**  **99**  **100**  **101**  **102**  **103**  **104**  **105**  **106**  **107**  **108**  **109**  **110**  **111**  **112**  **113**  **114**  **115**  **116**  **117**  **118**  **119**  **120**  **121**  **122**  **123**  **124**  **125**  **126**  **127**  **128**  **129**  **130**  **131**  **132**  **133**  **134**  **135**  **136**  **137**  **138**  **139**  **140** | ZmWRKY67  ZmWRKY68  ZmWRKY69  ZmWRKY70  ZmWRKY71.1  ZmWRKY71.2  ZmWRKY72  ZmWRKY73.1  ZmWRKY73.2  ZmWRKY74  ZmWRKY75.1  ZmWRKY75.2  ZmWRKY76  ZmWRKY77  ZmWRKY78.1  ZmWRKY78.2  ZmWRKY79  ZmWRKY80  ZmWRKY81  ZmWRKY82.1  ZmWRKY82.2  ZmWRKY83  ZmWRKY84.1  ZmWRKY84.2  ZmWRKY85  ZmWRKY86  ZmWRKY87  ZmWRKY88  ZmWRKY89  ZmWRKY90  ZmWRKY91  ZmWRKY92  ZmWRKY93  ZmWRKY94  ZmWRKY95  ZmWRKY96.1  ZmWRKY96.2  ZmWRKY97  ZmWRKY98  ZmWRKY99  ZmWRKY100  ZmWRKY101  ZmWRKY102  ZmWRKY103  ZmWRKY104  ZmWRKY105  ZmWRKY106*  ZmWRKY107  ZmWRKY108  ZmWRKY109.1  ZmWRKY109.2  ZmWRKY110  ZmWRKY111  ZmWRKY112  ZmWRKY113  ZmWRKY114  ZmWRKY115  ZmWRKY116.1  ZmWRKY116.2  ZmWRKY117  ZmWRKY118  ZmWRKY119  ZmWRKY120  ZmWRKY121.1  ZmWRKY121.2  ZmWRKY121.3  ZmWRKY122  ZmWRKY123  ZmWRKY124  ZmWRKY125 | Nucleus  Nucleus  Nucleus  Nucleus  Nucleus  Endoplasmic reticulum  Nucleus  Nucleus  Nucleus  Nucleus  Chloroplast  Endoplasmic reticulum  Cytoplasmic  Nucleus  Nucleus  Nucleus  Nucleus  Nucleus  Nucleus  Nucleus  Nucleus  Nucleus  Nucleus  Nucleus  Nucleus  Nucleus  Nucleus  Nucleus  Cytoplasmic  Nucleus  Nucleus  Nucleus  Nucleus  Nucleus  Nucleus  Mitochondria  Nucleus  Nucleus  Nucleus  Nucleus  Chloroplast  Chloroplast  Nucleus  Nucleus  Nucleus  Nucleus  Nucleus  Nucleus  Nucleus  Nucleus  Nucleus  Nucleus  Nucleus  Nucleus  Nucleus  Mitochondria  Nucleus  Nucleus  Nucleus  Nucleus  Nucleus  Nucleus  Nucleus  Chloroplast  Nucleus  Nucleus  Nucleus  Nucleus  Nucleus  Nucleus |
